# Synthesis, Spectroscopic, X-ray Diffraction and DFT Studies of Novel Benzimidazole Fused-1,4-Oxazepines

**DOI:** 10.3390/molecules21060724

**Published:** 2016-06-03

**Authors:** Abdulrahman I. Almansour, Natarajan Arumugam, Raju Suresh Kumar, Saied M. Soliman, Mohammad Altaf, Hazem A. Ghabbour

**Affiliations:** 1 Department of Chemistry, College of Science, King Saud University, P.O. Box 2455, Riyadh 11451, Saudi Arabia; almansor@ksu.edu.sa (A.I.A.); sraju@ksu.edu.sa (R.S.K.); 2 Department of Chemistry, College of Science & Arts, King Abdulaziz University, P. O. Box 344, Rabigh 21911, Saudi Arabia; saied1soliman@yahoo.com; 3 Department of Chemistry, Faculty of Science, Alexandria University, P.O. Box 426, Ibrahimia, Alexandria 21321, Egypt; 4 Central Laboratory, College of Science, King Saud University, P.O. Box 2455, Riyadh 11451, Saudi Arabia; altafamu@gmail.com; 5 Department of Pharmaceutical Chemistry, College of Pharmacy, King Saud University, P.O. Box 2457, Riyadh 11451, Saudi Arabia; ghabbourh@yahoo.com; 6 Department of Medicinal Chemistry, Faculty of Pharmacy, University of Mansoura, Mansoura 35516, Egypt

**Keywords:** 1,4-oxazepine, benzimidazole 2-carboxaldehyde, X-ray diffraction studies, DFT-calculation

## Abstract

A series of benzimidazole-tethered oxazepine heterocyclic hybrids has been synthesized in good to excellent yields from an *N*-alkylated benzimidazole 2-carboxaldehyde, which in turn was accomplished from *o*-phenylenediamine in three good yielding steps. The calculated molecular structure of compounds 2-methyl-4-(2-((phenylimino)methyl)-1*H*-benzo-[*d*]imidazol-1-yl)-butan-2-ol **9** and **10** 3,3-dimethyl-*N*-phenyl-1,2,3,5-tetrahydrobenzo-[4,5]imidazo[2,1-*c*][1,4]oxazepin-5-amine using the B3LYP/6–31 G(d, p) method were found to agree well with their X-ray structures. The charge distributions at the different atomic sites were computed using the natural bond orbital (NBO) method. The regions of electrophilic and nucleophilic reactivity were shown using a molecular electrostatic potential (MEP) map. In addition, the frontier molecular orbitals of these compounds were discussed at the same level of theory. Nonlinear optical (NLO) properties have also been investigated by computational hyperpolarizability studies, and it was found that Compound **9** is the best candidate for NLO applications.

## 1. Introduction

**Figure 1:**
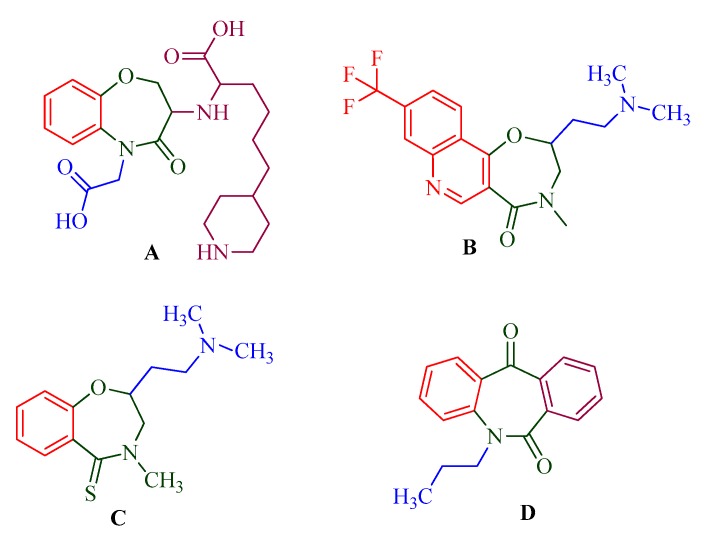
Promising biologically-active oxazepine leads.

The 1,4-oxazepine structural motif has gained potential importance in medicinal chemistry, as its derivatives exhibited a wide range of biological activities, such as effective protease inhibitors [1], non-peptidergic GPCR inhibitors [2], integrin antagonists [3], squalene synthase [4] and reverse transcriptase inhibitors [5]. For example, the oxazepin-4-one **A** (Figure 1) is a strong inhibitor of angiotensin-converting enzyme (ACE) [1] and found to play a prominent role in the current approaches to hypertension and diabetic nephropathy therapy, end-organ protection and heart failure treatment [6]. Oxazepine derivatives, *viz.* **B** and **C**, represent a promising class of compounds and are found to display H1 antihistaminic activity [7]. Furthermore, these compounds can also be used for the effective treatment of some allergic reactions including anticonvulsant [8], CNS depressant [9] and antifungal [10] activities. The *N*-substituted oxazepine derivative **D** is a selective inhibitor of human immunodeficiency virus type 1 (HIV-1) reverse transcriptase [11]. Moreover, its derivatives are exhibited as progesterone receptor agonists [12] and telomerase inhibitors [13]. Recently, Peng-Cheng Lv *et al.* [14] revealed that the oxazepine derivatives also act as selective inhibitors of PI3Kα for the treatment of cancer.

Benzimidazole is another important heterocyclic scaffold, as it possesses a wide range of biological activities, *viz*. antiviral [15], anti-inflammatory [16], anti-HIV-1 [17], antioxidant [18], antiproliferative, antitumor and potential anticancer activities [19,20,21,22]. Furthermore, the benzimidazole unit acts as an active moiety in many of the approved drugs, *viz*. omeprazole, lansoprazole, albendazole and oxfendazole. In recent years, we have been involved in the synthesis of diverse heterocyclic hybrids [23,24,25,26], including biologically-active benzimidazoles [27,28] through cycloaddition/straightforward multistep transformations.

In continuation of our earlier work towards the synthesis of diverse heterocycles in conjunction with the aforesaid biological significance of 1,4-oxazepine and benzimidazole scaffolds, herein we disclose our experimental and theoretical investigations on the synthesis and characterization of diverse benzimidazole-fused 1,4-oxazepine heterocyclic hybrids. Gaussian’03 software has been employed to perform the density functional theory (DFT) calculations to understand the electronic properties. The nonlinear optical activities of the synthesized compounds have also been investigated through computational hyperpolarizability studies.

## 2. Results and Discussion

### 2.1. Synthesis

The synthesis of hitherto unexplored benzimidazole-tethered 1,4-oxazepines was accomplished from benzimidazole 2-carboxaldehyde in the presence of the InCl_3_-catalyzed cyclization reaction. Our synthetic work began with an inexpensive precursor, *viz.* *o*-phenylenediamine **1**, which upon reaction with ethyl diethoxyacetate **2** furnished 2-diethoxymethyl-1*H*-benzimidazole **3** in an 80% yield [29,30]. The benzimidazole **3** was then subjected to *N*-alkylation using prenyl bromide **4** with sodium hydride in dry THF for 12 h to afford the *N*-alkylated benzimidazole **5**, which was confirmed through spectroscopic data.

**Scheme 1:**
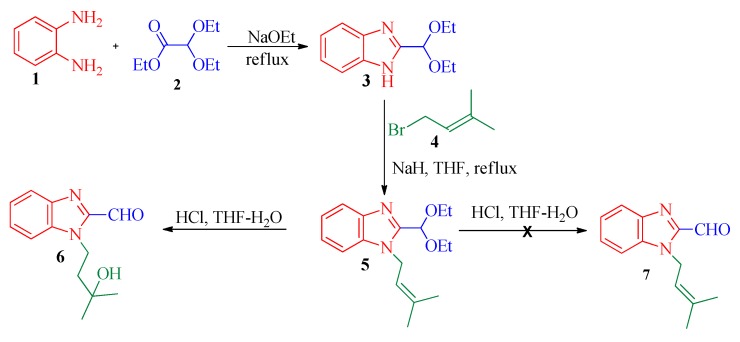
Synthesis of *N*-alkyl benzimidazole 2-carboxaldehyde.

Having synthesized the *N*-alkylated benzimidazole **5** (Scheme 1), the next task was to convert the acetal into an aldehyde in refluxing HCl/H_2_O in THF. This reaction in 24 h furnished the unusual 1-(3-hydroxy-3-methylbutyl)-benzimidazole carboxaldehyde **6** (Scheme 1), and the expected *N*-prenyl-benzimidazole carboxaldehyde **7** was not obtained, even in trace. The crude product **6** was purified by column chromatography and the structure of this unusual product was confirmed by spectroscopic studies. With 1-(3-hydroxy-3-methylbutyl)-benzimidazole carboxaldehyde **6** in hand, next, we turned our attention to the synthesis of 2-aryliminomethyl-(benzimidazol-1-yl)butan-2-ols **9a**–**e**. These are typically prepared by gentle mixing of corresponding benzimidazole carboxaldehyde **6** and arylamines **8a**–**e** under solvent-free conditions for 2–3 min in excellent yield (92%–97%). The imine **9** can be used as an attractive chelating agent, as it possesses two imino nitrogen atoms, which can readily bind with inorganic metals, leading to the formation of complex molecules.

**Scheme 2:**
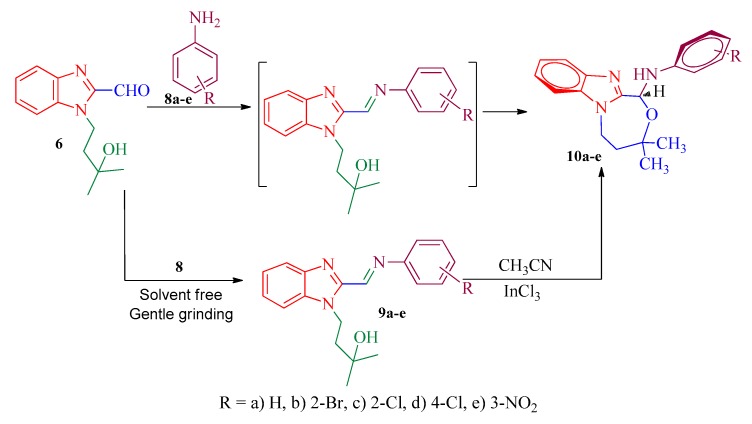
Synthesis of benzimidazole-fused 1,4-oxazepines.

**Figure 2:**
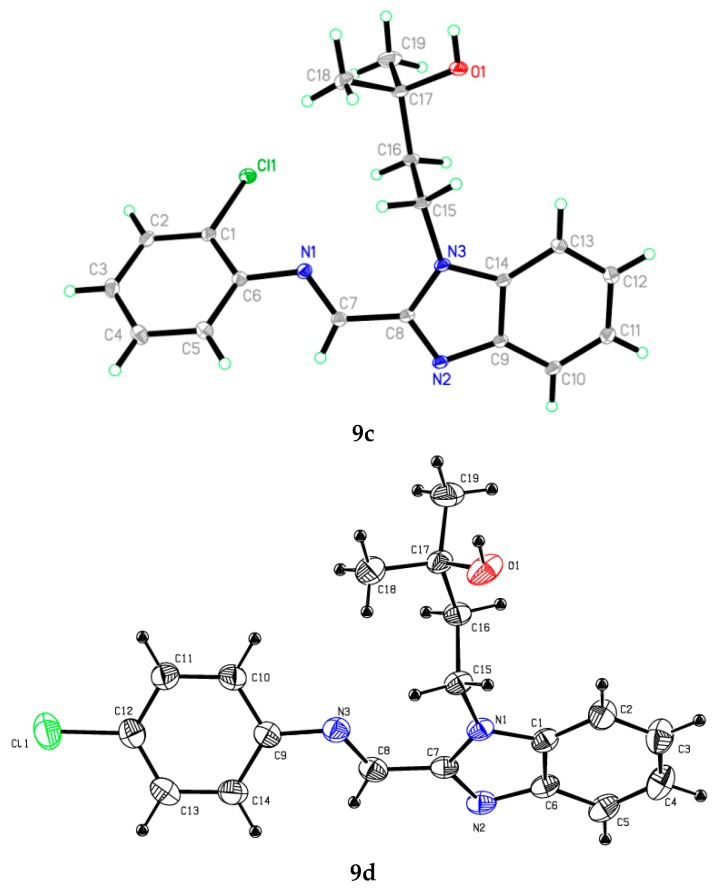
ORTEP diagram of **9c** and **9d**.

**Figure 3:**
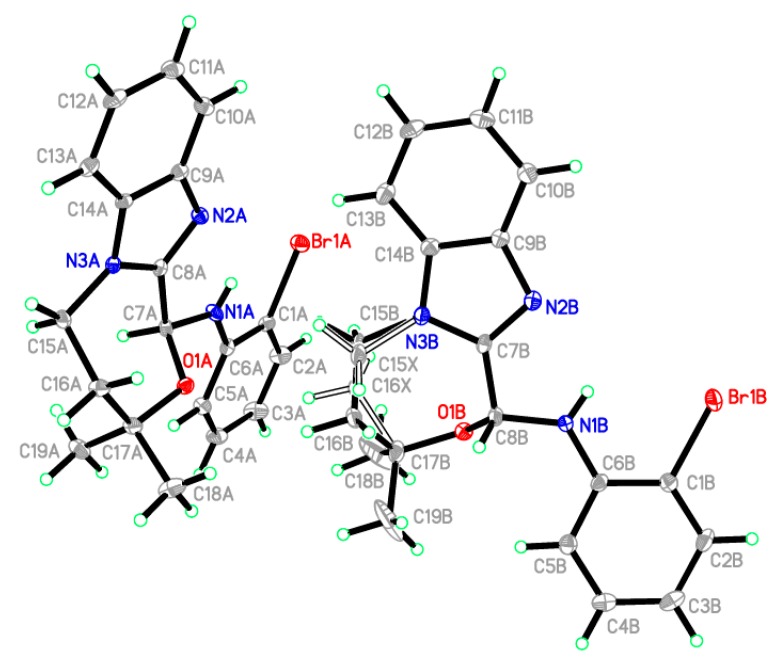
Diagram showing the ORTEP of **10b** with partial disorder in one of the molecules.

Initially, the reaction in CH_3_CN (3 mL) resulted in the formation of a yellow precipitate in a very short time (2–3 min). TLC analysis of this precipitate confirms the formation of a sole imine with good purity. The rapid formation of the product in the solution phase prompted us to attempt the reaction in the solid state with simple grinding without any solvent as an initiative to green synthesis. As expected, we observed the formation of **9a**–**e** in excellent yield (Table 1), this method gains importance, as the product can be obtained as such in pure form without any work up procedure. The structure of imine **9** was elucidated using ^1^H, ^13^C and mass spectroscopic data and was further confirmed unambiguously by a single crystal X-ray diffraction study of **9c** and **9d** (Figure 2) [31]. Having Compound **9** with flexible *N*-alkyl alcohol, we attempted its cyclisation with the imino carbon to afford the benzimidazole fused 1,4-oxazepines. Initially, the imine **9** in acetonitrile was stirred at ambient temperature. Even after a prolonged reaction time (72 h), the imine remained as such, and no reaction was observed. Later, the same reaction was refluxed for 12 h with regular monitoring (TLC analysis every 2 h), and we did not observe the formation of the desired product. The same reaction was then carried out in the presence of InCl_3_ at ambient temperature overnight, and we observed the formation of the unusual product **10** in moderate yields. In an attempt to improve the yield, the reaction was performed under reflux in acetonitrile in the presence of InCl_3_. This reaction afforded the unusual Compound **10** in good yields. The cyclisation reactions were also investigated with various Lewis acids, such as BF_3_·Et_2_O, Yb(OTf)_3_ and SC(OTf)_3_, and found an inseparable mixture of compounds in TLC analysis. The reaction was also performed with high boiling solvents, such as DMF, toluene, xylene and dichlorobenzene. The starting materials were undiminished even after 72 h. Later, on the addition of 20% InCl_3_, the reaction resulted in the formation of inseparable mixture of spots, as evidenced by TLC analysis. The cyclisation reaction of Compound **9** bearing electron-donating groups, such as *p*-methoxy and *p*-methyl, failed even after prolonged reaction time (72 h), probably due to the more electron-rich imine, which prevents the cyclisation. As a more efficient alternative, the preparation of heterocyclic hybrid **10** through a one-pot cyclization reaction without isolation of imine was sought. The desired reaction was carried out successfully by treating an equimolar amount of benzimidazole carboxaldehyde **6** and various substituted arylamines **9** in CH_3_CN under reflux in the presence of InCl_3_, furnishing the target benzimidazole fused 1,4-oxazepines **10** in good yields (Scheme 2) (Table 1). The structure of Compound **10** was fully characterized by ^1^H and ^13^C, mass and elemental analysis. Finally, the stereochemistry of the heterocyclic hybrid was unambiguously confirmed by single crystal X-ray diffraction analysis [32] of **10b** (Figure 3).

**Scheme 3:**
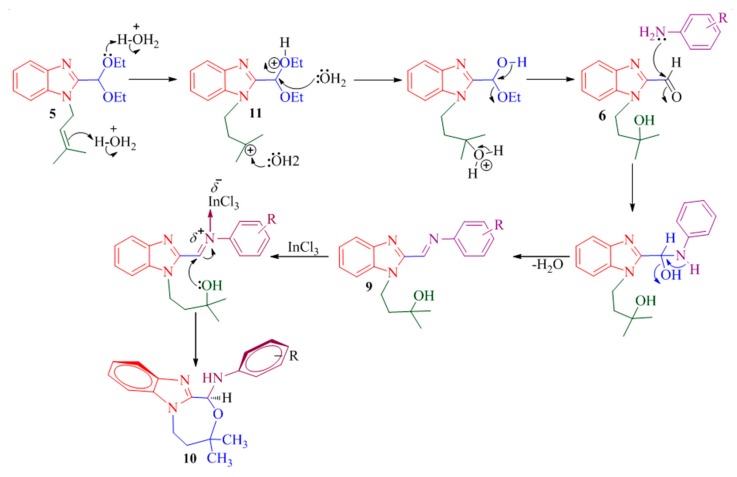
Plausible mechanism for the formation of benzimidazole-fused 1,4-oxazepines.

A feasible mechanism for the formation of benzimidazole fused 1,4-oxazepine **10** is described in Scheme 3. Compound **5** was protonated to generate tertiary carbocation **11**, which then was attacked by water molecule to furnish benzimidazole 2-carboxaldehyde **6**. Compound **6** further reacts with arylamine to afford novel imino benzimidazole alcohol **9**. The imino carbon of **9** was attacked by a lone pair electron of alcohol through intramolecular nucleophilic addition to furnish Compound **10** in excellent yields.

### 2.2. X-ray Crystallographic Studies

A crystal with a yellow color and a block shape of C_19_H_20_ClN_3_O was selected for single crystal X-ray analysis. A total of 1858 frames were collected in 4.76 h. The Bruker SAINT software package was used in the integration of the frames. The data were integrated using an orthorhombic unit cell with 44,126 reflections to θ_max_ 33.80° (0.64 Å resolution), of which 7032 were independent (average redundancy 6.275, completeness = 99.8%, R_int_ = 2.64%, R_sig_ = 2.11%). The final cell constants of *a* = 18.3707(5) Å, *b* = 6.3836(2) Å, *c* = 15.0003(4) Å and volume = 1759.10(9) Å^3^ are based on the refinement of the XYZ-centroids of 9919 reflections above 20 σ(I) with 5.431° < 2θ < 67.52°. The multi-scan method (SADABS) was used for the correction of absorption.

The molecules **9c** and **9d** are composed of a benzimidazole ring with a 2-aryliminomethyl group (Figure 1), and the dihedral angles between the two rings are 52.68(2)° and 39.05(2)° for Compounds **9c** and **9d**, respectively. The torsion angle in between C11–N2–C7–C9 is 40.0(2)°. There are two intramolecular interactions between C15–H15B···N3 and C15–H15B···N1 in Compound **9c** and C15—H15A···O1 and C15–H15B···N3 in Compound **9d**. In the crystal structure, two intermolecular O1–H3···N2 and C2–H2···O1 hydrogen bonds are observed in Compound **9d**, but only one intermolecular O1–H1···N2 in Compound **9d**. The selected geometric parameters and distances of the donor–H, acceptor/H, donor/acceptor and donor–H/acceptor angles are presented in the Supplementary Materials.

In the crystal structure of Compound **10b**, the unit cell was composed of two independent molecules (Figure 2); one of them has a partial disorder in the 1,4-oxazepines ring; the disorder ratio refined to 0.59094 (5): 0.40906 (8). There are no significant differences between bond lengths and angles in the two structures. The seven-membered oxazepine ring (O1/N3/C7/C8/C15–C17) has a twist-chair conformation, and the phenyl ring makes a dihedral angle of 8.92(3)° and 12.92(4)° with the benzimidazole ring system mean plane in the molecules **A** and **B**, respectively, which differ from the similar reported compounds, which make much larger angles reach 73.42(10)° and 83.07(7)° [33]. In the crystal packing, molecules are linked via one intermolecular hydrogen bond between C8B–H8BA···N2B^i^ with symmetry code: (i) −*x* + 1, −*y* + 2, −*z* + 1. The selected geometric parameters and distances of the donor–H, acceptor/H, donor/acceptor and donor–H/acceptor angles are presented in the Supplementary Materials.

### 2.3. Optimized Molecular Geometry

**Figure 4:**
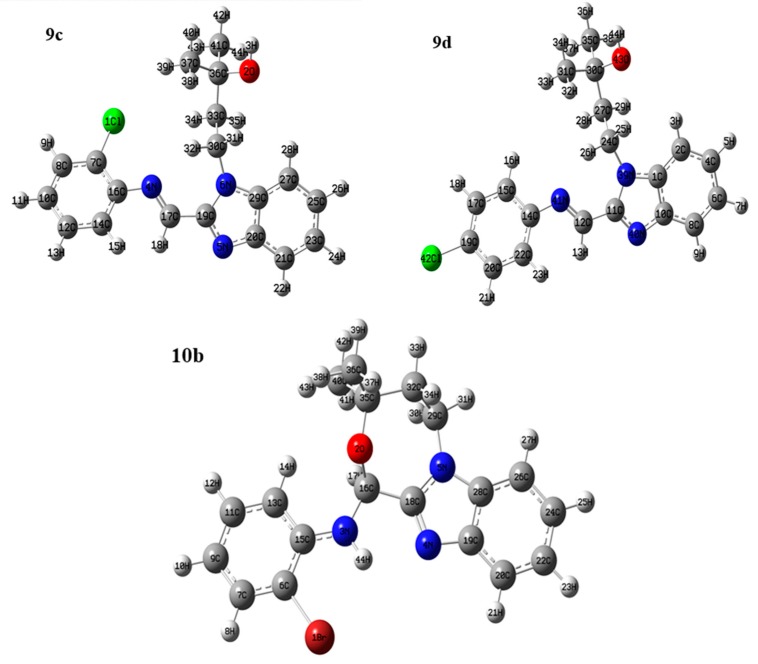
The optimized molecular structure of the studied compounds.

The optimized structures of Compounds **9c**, **9d** and **10b** are given in Figure 4. All of the structures have a C_1_ point group. The optimized geometric parameters are given in the Supplementary Materials. The calculated and experimental bond distances and bond angles are in good agreement with each other. The calculated root mean square deviations (RMSD) of the bond distances and bond angles are in the range of 0.009–0.018 Å and 0.4–0.7°, respectively. Moreover, good correlations (*R*^2^ = 0.994–0.997) between the calculated and experimental data were obtained. In general, the bond distances and bond angles are predicted very well.

### 2.4. Natural Atomic Charges

The dipole moment, molecular polarizability and electronic structure of a compound are dependent on the atomic charge distribution. The calculated charge populations using the NBO method are given in the supplementary data. The *O*- and *N*-atoms have the highest electronegative charges among the different atomic sites. The charge values at the N4 (−0.4553), N41 (−0.4620) and N3 (−0.6350) for Compounds **9c**, **9d** and **10b** respectively showed that the N-atom of the amino (NH) group of **10b** has higher negative charge density than that for the imine (C=N) moiety of the others. This could be explained due to the change in the hybridization of this N-atom due to the cyclization. Moreover, the two benzimidazole N-atoms have the most negative natural charges for **10b**. In general, the N-atom attached to the alkyl substituent has less negative charge density than the free one for all compounds. For **9c** and **9d**, the atomic charges at the O- and N-sites are affected very slightly by changing the position of the Cl-substituent at the phenyl ring. The aliphatic C-atoms have more negative charge than the aromatic ones where the C-atoms attached to either O or N are electropositive. The dipole moment values of **9c**, **9d** and **10b** are 2.79, 3.92 and 5.77 Debye, respectively. From this point of view, one could conclude that the compound with para-Cl substitution is more polar than the ortho-Cl one. Furthermore, the cyclic adduct has the highest polarity of these compounds.

**Figure 5:**
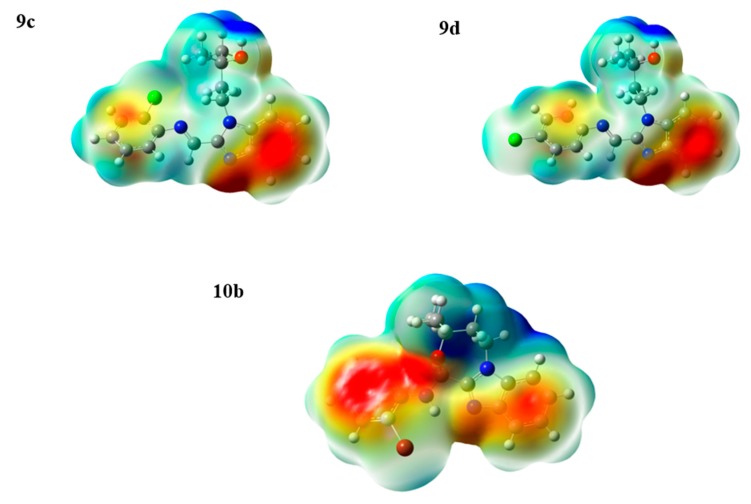
Molecular electrostatic potentials (MEP) mapped on the electron density surface calculated by the DFT/B3LYP method.

The molecular electrostatic potential (MEP) is a common map used to visualize the reactive sites for electrophilic and nucleophilic attack. Furthermore, it is used in studies of biological recognition and hydrogen bonding interactions [34,35]. The MEP figures of **9c**, **9d** and **10b** at the same level of theory are shown in Figure 5. It can be seen from this figure that negative regions (red) are mainly localized over the unsubstituted N-atom of the benzimidazole moiety and the O-atom of the OH group. Hence, in the studied compounds, these atoms are the most reactive sites for electrophilic attack, as well as the more proper sites to attack the positive regions of the receptor molecule. In contrast, the maximum positive regions (blue) are localized over the H-atom of the OH group in the case of **9**, which is the most reactive sites for nucleophilic attack.

### 2.5. Frontier Molecular Orbitals

**Figure 6:**
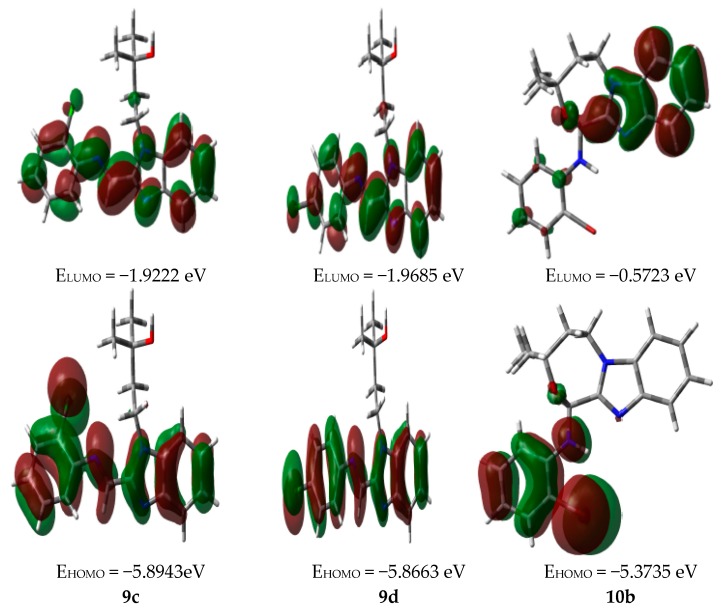
The ground state isodensity surface plots for the frontier molecular orbitals.

The HOMO and LUMO levels, which are called frontier molecular orbitals (FMOs), are important for chemists and physical chemists [36]. Their energies and electron densities are important to describe the chemical reactivity and excitation properties. Furthermore, the energy gap between HOMO and LUMO (ΔE) gives the lowest energy for electronic transition. Moreover, the HOMO-LUMO intramolecular charge transfer (ICT) has an application to prove the bioactivity [37,38]. The ΔE value represents the lowest energy for ICT where the E_HOMO_ and E_LUMO_ of the studied molecules are calculated using the B3LYP/6–31 G(d, p) method. The HOMO and LUMO pictures are shown in Figure 6. In the studied compounds, the HOMO–LUMO energy gaps are 3.9721, 3.8978 and 4.8012eV for **9c**, **9d** and **10b**, respectively. This intramolecular charge transfer belongs mainly to π-π* excitations. The HOMO and LUMO of Compounds **9**, are mainly localized over the π-system of the benzimidazole and phenyl rings. For **10b**, the HOMO is localized over the phenyl ring, while the LUMO is localized over the benzimidazole moiety. The HOMO→LUMO electronic transition energies of the studied compounds indicated the greater difficult of ICT for Compound **10b**.

### 2.6. Nonlinear Optical Properties

Nonlinear optical materials have many industrial and medical applications [39]. It is important for photonic instruments in which light is used for data transmission rather than electrons. Hyperpolarizability (β) is one significance parameter for describing the nonlinear optical properties of molecular systems. The calculated values of hyperpolarizability components and the average hyperpolarizability (β_0_) are of the studied compounds, as well as urea, which is a well-known reference [40] for comparison purposes of nonlinear optical properties (NLO) activity, and are given in the Supplementary Data. The calculated hyperpolarizability (β_0_) of Compounds **9c**, **9d** and **10b** are 2307.823, 958.248 and 407.879 a.u., respectively, while for urea, it is 69.914 a.u. These results indicated that for all of the studied compounds, **9c**, **9d** and **10b** have hyperpolarizability (β_0_) that is higher up to 33-, 14- and 6-times, respectively, than urea. The best is for **9c**, which could be predicted as the best candidate for NLO application.

## 3. Materials and Methods

### 3.1. General Procedure

The melting point was taken using an open capillary tube and is uncorrected. Unless stated otherwise, solvents and chemicals were obtained from commercial sources and used without further purification. ^1^H- and ^13^C-NMR spectra were recorded on a Varian Mercury JEOL-400 NMR spectrometer (Tokyo, Japan) and Bruker 500 MHz NMR spectrometers (Faellanden, Switzerland) operating at 400, 500, 100 and 125 MHz, respectively, and chemical shifts are reported as δ values (ppm) relative to tetramethylsilane. Elemental analyses were performed on a Perkin-Elmer 2400 series II elemental CHNS analyzer (Waltham, MA, USA).

### 3.2. Synthesis of 2-(Diethoxymethyl)-1-(3-methylbut-2-en-1-yl)-1H-benzimidazole, ***5***

2-(diethoxymethyl)-*1H*-benzimidazole **3** (3 g, 15.4 mmol) was added to NaH (630 mg, 26.3 mmol) in THF (300 mL) and refluxed for 30 min. 1-bromo-3-methylbut-2-ene (3.45 g, 23.2 mmol) was added and the resulting solution refluxed for a further 8 h. TLC of the reaction mixture showed that the starting material was completely converted to product. The mixture was cooled to room temperature, and water (100 mL) was added. The product was extracted with Et_2_O (2 × 75 mL), dried (MgSO_4_), filtered and evaporated to dryness to yield sole 2-(diethoxymethyl)-1-(3-methylbut-2-en-1-yl)-1*H*-benzimidazole **5** as a brown viscous oil, and the crude product was purified by column chromatography. Spectroscopic data for **5**: ^1^H-NMR (400 MHz, CDCl_3_) δ_H_: 1.17 (t, 6H), 1.65 (s, 3H), 1.80 (s, 3H), 3.48–3.54 (m, 2H), 3.67–3.73 (m, 2H), 4.94 (d, *J* = 5.88 Hz, 2H), 5.21–5.23 (m, 1H) 5.65 (s, 1H), 7.14–7.22 (m, ArH, 3H), 7.69–7.71 (m, Ar–H, 1H) ppm; ^13^C-NMR (100 MHz, CDCl_3_) δ_C_: 14.7, 17.9, 25.3, 42.3, 63.0, 98.9, 109.9, 119.7, 119.9, 121.7, 122.7, 134.7, 135.4, 141.7, 149.9. Anal. Calcd. For C_17_H_24_N_2_O_2_: C, 70.80; H, 8.39; N, 9.71%. Found: C, 70.93; H, 8.50; N, 9.83%.

### 3.3. Synthesis of 1-Allyl-1H-benzimidazole-2-carboxaldehydes, ***6***

A mixture of 2-(diethoxymethyl)-1-(3-methylbut-2-en-1-yl)-1*H*-benzimidazole **5** (3.0 g, 10.98 mmol), water (18 mL), 36% hydrochloride acid (9 mL) and THF (50 mL) were refluxed for 24 h. Then, the reaction mixture was neutralized with saturated sodium bicarbonate and extracted with EtOAc (3 × 70 mL). The extract was washed with brine (2 × 50 mL), dried, filtered and evaporated. The crude product was purified by column chromatography. 1-(3-hydroxy-3-methylbutyl)-1*H*-benzimidazole-2-carbaldehyde **6**: Colorless oil. ^1^H-NMR (500 MHz, CDCl_3_) δ_H_: 1.37 (s, 6H), 1.92–1.96 (m, 2H), 4.71–4.74 (m, 2H), 7.16–7.53 (m, ArH, 3H), 7.87–7.93 (m, 1H), 10.08 (s, 1H, CHO) ppm; ^13^C-NMR (125 MHz, CDCl_3_) δ_C_: 30.2, 40.9, 43.2, 69.98, 110.9, 122.3, 124.1, 126.8, 136.1, 142.8, 145.8, 184.9. Anal. Calcd. For C_13_H_16_N_2_O_2_: C, 67.22; H, 6.94; N, 12.06%. Found: C, 67.34; H, 6.85; N, 12.17%.

### 3.4. General Procedure for the Synthesis of (E)-2-Methyl-4-(2-((phenylimino)methyl)-1H-benzimidazol-1-yl)butan-2-ol, ***9a**–**e***

A mixture of benzimidazole carboxaldehyde **6** (100 mg, 0.43 mmol) and aniline **8** (0.43 mmol) was ground well in a mortar at ambient temperature for 2–3 min. The completion of the reaction was evidenced through the formation of a yellow precipitate, which was then washed with water and dried under vacuum. TLC analysis of the yellow precipitate confirms the formation of the imine in a good yield.

*(E)-2-methyl-4-(2-(phenylimino)methyl)-1H-benzimidazol-1-yl)butan-2-ol*, **9a:** Yellow solid. ^1^H-NMR (400 MHz, CDCl_3_) δ_H_: 1.35 (s, 6H), 2.09–2.13 (m, 2H), 4.94–4.98 (m, 2H), 7.22–7.88 (m, ArH, 9H), 8.80 (s, 1H) ppm; ^13^C-NMR (100 MHz, CDCl_3_) δ_C_: 30.4, 41.2, 42.7, 70.2, 110.3, 121.1, 121.2, 123.4, 125.2, 129.6, 136.4, 137.7, 143.6, 146.9, 148.6, 152.6. LC/MS (ESI): *m*/*z* 307 [M]^+^. Anal. Calcd. For C_19_H_21_N_3_O: C, 74.24; H, 6.89; N, 13.67%. Found: C, 74.37; H, 6.77; N, 13.55%.

*(E)-4-(2-(((2-bromophenyl)imino)methyl)-1H-benzimidazol-1-yl)-2-methylbutan-2-ol*, **9b:** Yellow solid. ^1^H-NMR (400 MHz, CDCl_3_) δ_H_: 1.35 (s, 6H), 2.13–2.17 (m, 2H), 4.98–5.02 (m, 2H), 7.25–7.87 (m, ArH, 8H), 8.62 (s, 1H) ppm; ^13^C-NMR (100 MHz, CDCl_3_) δ_C_: 29.9, 41.7, 43.1, 70.7, 110.5, 117.21, 119.7, 121.5, 123.3, 123.6, 125.7, 130.1, 130.2 136.6, 143.4, 146.6, 151.8, 153.9. LC/MS (ESI): *m*/*z* 386 [M]^+^. Anal. Calcd. For C_19_H_20_BrN_3_O: C, 59.08; H, 5.22; N, 10.88%. Found: C, 59.21; H, 5.35; N, 10.99%.

*(E)-4-2-(2-chlorophenylimino)methyl)-1H-benzimidazol-1-yl)-2-methylbutan-2-ol*, **9c:** Yellow solid. ^1^H-NMR (400 MHz, CDCl_3_) δ_H_: 1.34 (s, 6H), 2.12–2.16 (m, 2H), 4.96–5.00 (m, 2H), 7.16–7.87 (m, ArH, 8H), 8.66 (s, 1H) ppm; ^13^C-NMR (100 MHz, CDCl_3_) δ_C_: 29.7, 41.5, 42.9, 70.2, 110.5, 119.5, 120.9, 123.1, 123.4, 125,4, 127.9, 128.2, 130.3, 136.6, 143.2, 146.9, 148.3, 153.3. LC/MS (ESI): *m*/*z* 342 [M]^+^. Anal. Calcd. For C_19_H_20_ClN_3_O: C, 66.76; H, 5.90; N, 12.29%; Found: C, 66.89; H, 5.77; N, 12.38%.

*(E)-4-2-(4-chlorophenylimino)methyl)-1H-benzimidazol-1-yl)-2-methylbutan-2-ol*, **9d:** Pale Yellow solid. ^1^H-NMR (400 MHz, CDCl_3_) δ_H_: 1.31 (s, 6H), 2.07–2.11 (m, 2H), 4.91–4.95 (m, 2H), 7.25–7.86 (m, ArH, 8H), 8.74 (s, 1H) ppm; ^13^C-NMR (100 MHz, CDCl_3_) δ_C_: 29.8, 41.3, 42.9, 70.1, 110.2, 121.1, 121.3, 122.3, 122.6, 129.6, 129.9, 133.3, 143.3, 146.9, 148.7, 153.0. LC/MS (ESI): *m*/*z* 342 [M]^+^. Anal. Calcd. For C_19_H_20_ClN_3_O: C, 66.76; H, 5.90; N, 12.29%; Found: C, 66.87; H, 5.79; N, 12.35%.

*(E)-2-methyl-4-(2-(((3-nitrophenyl)imino)methyl)-1H-benzimidazol-1-yl)butan-2-ol*, **9e:** Pale Yellow solid. ^1^H-NMR (400 MHz, CDCl_3_) δ_H_: 1.37 (s, 6H), 2.08–2.16 (m, 2H), 4.91–4.93 (m, 2H), 7.18–7.91 (m, ArH, 8H), 8.74 (s, 1H) ppm; ^13^C-NMR (100 MHz, CDCl_3_) δ_C_: 29.8, 41.3, 42.9, 70.1, 110.4, 117.1, 121.2, 123.2, 123.4, 124.2, 125.5, 130.3, 130.9, 136.5, 143.3, 146.7, 151.7, 153.8. LC/MS (ESI): *m*/*z* 352 [M]^+^. Anal. Calcd. For C_19_H_20_N_4_O_3_: C, 64.76; H, 5.72; N, 15.90; %. Found: C, 64.85 H, 5.81; N, 15.78%.

### 3.5. General Procedure for Synthesis of Benzimidazole Fused 1,4-Oxazepines, ***10a**–**e***

A mixture of benzimidazole carboxaldehyde **6**, arylamine **8** and 20 mol% InCl_3_ in acetonitrile was refluxed overnight. After completion of the reaction as evidenced by TLC analysis, the reaction mixture was concentrated under reduced pressure. The crude product was purified by column chromatography. The product was crystallized by EtOAc by the slow evaporation technique.

*3,3-Dimethyl-N-phenyl-1,2,3,5-tetrahydrobenzo[4,5]imidazo[2,1-c][1,4]oxazepin-5-amine*, **10a**: White solid. ^1^H-NMR (400 MHz, CDCl_3_) δ_H_: 1.32 (s, 6H), 1.83–1.88 (m, 2H), 3.54–3.56 (m, 1H), 3.63–3.65 (m, 1H), 4.33 (s, 1H), 7.25–7.85 (m, ArH, 9H) ppm; ^13^C-NMR (100 MHz, CDCl_3_) δ_C_: 29.8, 40.3, 42.5, 71.0, 75.9, 110.3, 114.2, 120.5, 121.4, 122.8, 123.1, 129.6, 136.3, 143.5, 146.4, 149.7. LC/MS (ESI): *m*/*z* 307 [M]^+^. Anal. Calcd. For C_19_H_21_N_3_O: C, 74.24; H, 6.89; N, 13.67%. Found: C, 74.37; H, 6.76; N, 13.80%.

*N-(2-Bromophenyl)-3,3-dimethyl-1,2,3,5-tetrahydrobenzo[4,5]imidazo[2,1-c][1,4]oxazepin-5-amine*, **10b**: Colorless crystals. ^1^H-NMR (400 MHz, CDCl_3_) δ_H_: 1.34 (s, 6H), 1.82–1.87 (m, 2H), 3.54–3.56 (m, 1H), 3.63–3.65 (m, 1H), 4.32 (s, 1H), 7.22–7.67 (m, ArH, 8H) ppm; ^13^C-NMR (100 MHz, CDCl_3_) δ_C_: 29.5, 41.4, 43.0, 71.0, 75.8, 110.5, 115.8, 118.8, 119.4, 122.5, 123.4, 124.1, 125.4, 128.9, 133.3, 143.3, 146.8, 149.8. LC/MS (ESI): *m*/*z* 386 [M]^+^. Anal. Calcd. For C_19_H_20_BrN_3_O: C, 59.08; H, 5.22; N, 10.88%. Found: C, 59.21; H, 5.35; N, 10.75%.

*N-(2-Chlorophenyl)-3,3-dimethyl-1,2,3,5-tetrahydrobenzo[4,5]imidazo[2,1-c][1,4]oxazepin-5-amine*, **10c**: White solid. ^1^H-NMR (400 MHz, CDCl_3_) δ_H_: 1.32 (s, 6H), 1.84–1.86 (m, 2H), 3.53–3.56 (m, 1H), 3.61–3.63 (m, 1H), 4.32 (s, 1H), 7.25–7.87 (m, Ar–H, 8H) ppm; ^13^C-NMR (100 MHz, CDCl_3_) δ_C_: 29.3, 39.8, 41.7, 71.5, 75.8, 110.2, 115.2, 119.3, 123.2, 123.3, 123.8, 124.4, 127.4, 131.6, 136.5, 141.7, 146.2, 148.3. LC/MS (ESI): *m*/*z* 342 [M]^+^. Anal. Calcd. For C_19_H_20_ClN_3_O: C, 66.76; H, 5.90; N, 12.29%. Found: C, 66.89; H, 5.79; N, 12.38%.

*N-(4-Chlorophenyl)-3,3-dimethyl-1,2,3,5-tetrahydrobenzo[4,5]imidazo[2,1-c][1,4]oxazepin-5-amine*, **10d**: White solid. ^1^H-NMR (400 MHz, CDCl_3_) δ_H_: 1.29 (s, 6H), 1.81–1.85 (m, 2H), 3.51–3.54 (m, 1H), 3.60–3.63 (m, 1H), 4.34 (s, 1H), 7.24–7.85 (m, ArH, 8H) ppm; ^13^C-NMR (100 MHz, CDCl_3_) δ_C_: 29.2, 39.8, 41.2, 71.6, 75.9, 110.0, 119.1, 120.2, 122.9, 123.2, 125.8, 129.2, 136.7, 141.9, 146.1, 148.4. LC/MS (ESI): *m*/*z* 342 [M]^+^. Anal. Calcd. For C_19_H_20_ClN_3_O: C, 66.76; H, 5.90; N, 12.29%. Found: C, 66.89; H, 5.79; N, 12.41%.

*3,3-Dimethyl-N-(3-nitrophenyl)-1,2,3,5-tetrahydrobenzo[4,5]imidazo[2,1-c][1,4]oxazepin-5-amine*, **10e**: White solid. ^1^H-NMR (400 MHz, CDCl_3_) δ_H_: 1.35 (s, 6H), 1.81–1.87 (m, 2H), 3.54–3.56 (m, 1H), 3.61–3.63 (m, 1H), 4.33 (s, 1H), 7.21–7.84 (m, ArH, 8H) ppm; ^13^C-NMR (100 MHz, CDCl_3_) *δ_C_*: 29.4, 39.3, 40.1, 71.6, 75.6, 107.6, 110.5, 113.8, 120.1, 121.3, 122.7, 123.4, 123.6, 129.7, 136.1, 141.3, 146.2, 149.3. LC/MS (ESI): *m*/*z* 352 [M]^+^. Anal. Calcd. For C_19_H_20_N_4_O_3_: C, 64.76; H, 5.72; N, 15.90%. Found: C, 64.88; H, 5.61; N, 15.79%.

### 3.6. Computational Details

All theoretical calculations of the above-reported compound have been investigated through the DFT method with the B3LYP functional and 6‒31 G(d, p) basis set using Gaussian 03 software [41]. The single crystal X-ray structure coordinates were used as starting point for the calculations. No symmetry restrictions were applied, and optimized structures were taken as the input for frequency calculations. No imaginary vibrations were detected; hence, the real energy minimum was obtained. GaussView4.1 [42] is used to visualize the optimized structures and to draw the frontier molecular orbitals (FMO), as well as the molecular electrostatic potential maps (MEP).

## 4. Conclusions

In conclusion, we describe a general and efficient approach for the synthesis of hitherto unexplored benzimidazole-fused 1,4-oxazepines in good yields from unusual 1-(3-hydroxy-3-methylbutyl)-benzimidazole carboxaldehyde via an unusual cyclization in the presence of InCl_3_. The molecular structure of the studied compounds has been optimized using the DFT/B3LYP method and 6–31 G(d, p) basis set. The calculated bond distances and bond angles showed good agreement with our reported X-ray crystal structures. The atomic charges, molecular electrostatic potential (MEP) map, as well as the HOMO and LUMO level analyses were discussed. Based on hyperpolarizability calculations, Compound **9c** is the best candidate for NLO applications.

## Figures and Tables

**Table 1:** Synthesis of benzimidazole Schiff base (**9a**–**e**) and benzimidazole fused 1,4-oxazepines (**10a**–**e**).

Entry	Imine	Mp °C	Yield ^a^ %	Product	Mp °C	Yield ^b^ %
1	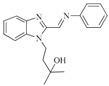 **9a**	155–157	91	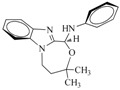 **10a**	197–199	60
2	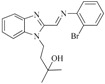 **9b**	234–236	93	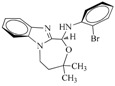 **10b**	220–222	63
3	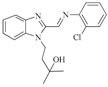 **9c**	241–243	92	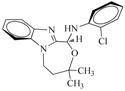 **10c**	225–227	62
4	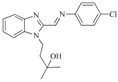 **9d**	180–182	96	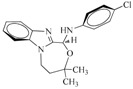 **10d**	252–254	64
5	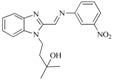 **9e**	188–190	90	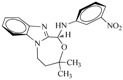 **10e**	222–224	67

^a^ Isolated yield by simple grinding; ^b^ isolated yield after purification by column chromatography.

## Supplementary Materials

Supplementary materials can be accessed at: http://www.mdpi.com/1420-3049/21/6/724/s1.

## References

[1] Robl J.A., Simpkins L.M., Asaad M.M. (2000). *N*-Formyl hydroxylamine containing dipeptides: Generation of a new class of vasopeptidase finhibitors. Bioorg. Med. Chem. Lett..

[2] Murakami Y., Hara H., Okada T., Hashizume H., Kii M., Ishihara Y., Ishikawa M., Shimamura M., Mihara S., Kato G. (1999). 1,3-Disubstituted benzazepines as novel, potent, selective neuropeptide Y Y1 receptor antagonists. J. Med. Chem..

[3] Fukui H., Ikegami S., Watanuki M., Maruyama T., Sumita Y., Inoguchi K. (2001). Azepine Derivatives. Patent WO.

[4] Yukimasa H., Tozawa R., Kori M., Kitano K. (1998). 4,1-benzoxazepin Derivatives and Their Use. U.S. Patent.

[5] Rodgers J.D., Cocuzza A.J. (2000). 5,5-disubstituted-1,5-dihydro-4,1-benzoxazepin-2(3*H*)-ones Useful as HIV Reverse Transcriptase Inhibitors. U.S. Patent.

[6] Sica D.A. (2005). Alpha1-adrenergic blockers: Current usage considerations. J. Clin. Hypertens..

[7] Cale A.D., Gero T.W., Walker K.R., Lo Y.S., Welstead W.J., Jaques L.W., Johnson A.F., Leonard C.A., Nolan J.C., Johnson D.N. (1989). Benzo- and pyrido-1,4-oxazepin-5-ones and -thiones: Synthesis and structure-activity relationships of a new series of H1-antihistamines. J. Med. Chem..

[8] Deng X.-Q., Wei C.-X., Li F.-N., Sun Z.-G., Quan Z.-S. (2010). Design and synthesis of 10-alkoxy-5, 6-dihydro-triazolo[4,3-*d*]benzo[f][1,4]oxazepine derivatives with anticonvulsant activity. Eur. J. Med. Chem..

[9] Kapples K.J., Effland R.C. (1993). Pyrrolo[2,1-*c*][1,4]benzoxazepines. 2. Synthesis of 5-phenyl derivatives. J. Heterocycl. Chem..

[10] Serrano-Wu M.H., St Laurent D.R., Chen Y., Huang S., Lam K.-R., Matson J.A., Mazzucco C.E., Stickle T.M., Tully T.P., Wong H.S. (2002). Sordarin Oxazepine Derivatives as Potent Antifungal Agents. Bioorg. Med. Chem. Lett..

[11] Klunder J.M., Hargrave K.D., West M., Cullen E., Pal K., Behnke M.L., Kapadia S.R., McNeil D.W., Wu J.C., Chow G.C. (1992). Novel non-nucleoside inhibitors of HIV-1 reverse transcriptase. 2. Tricyclic pyridobenzoxazepinones and dibenzoxazepinones. J. Med. Chem..

[12] Paul P.M.A.D., Brigitte J.B.F., Hans H., Cor W.K., Hans L., Lourdes O., Jos B.M.R., Pedro H.H.H. (2008). SAR study of 2,3,4,14b-tetrahydro-1*H*-dibenzo[*b*,*f*]pyrido[1,2-*d*][1,4]oxazepines as progesterone receptor agonists. Bioorg. Med. Chem. Lett..

[13] Xin-Hua L., Ying-Ming J., Bao-An S., Zhi-xiang P., Song Y. (2013). Design and synthesis of novel 2-methyl-4,5-substitutedbenzo[*f*]-3,3a,4,5-tetrahydro-pyrazolo[1,5-*d*][1,4]oxazepin-8(7*H*)-one derivatives as telomerase inhibitors. Bioorg. Med. Chem. Lett..

[14] Yong Y., Yan-Qing Z., Biao J., Shao S., Xun W., Chetan B.S., She-Feng W., Fang Q., Ai-Min Lu., Peng-Cheng L. (2015). 6,7-Dihydrobenzo[*f*]benzo[4,5]imidazo[1,2-*d*][1,4]oxazepine derivatives as selective inhibitors of PI3Kα. Bioorg. Med. Chem..

[15] Komazin G., Ptak R.G., Emmer B.T., Townsend L.B., Drach J.C. (2003). Resistance of Human Cytomegalovirus to the Benzimidazole l-Ribonucleoside Maribavir Maps to UL27. Nucleosides Nucleotides. Nucl..

[16] Mader M., De Dios A., Shih C., Bonjouklian R., Li T., White W., López de Uralde B., Sánchez-Martinez C., Prado M.D., Jaramillo C. (2008). Imidazolyl benzimidazoles and imidazo[4,5-*b*]pyridines as potent p38α MAP kinase inhibitors with excellent *in vivo* antiinflammatory properties. Bioorg. Med. Chem. Lett..

[17] Rao A., Chimirri A., Clercq E.D., Monforte A.M., Monforte P., Pannecouque C., Zappala M. (2002). Synthesis and anti-HIV activity of 1-(2,6-difluorophenyl)-1*H*,3*H*-thiazolo[3,4-a]benzimidazole structurally-related 1,2-substituted benzimidazoles. II Farmaco.

[18] Goker H., Kus C., Boykin D.W., Yildiz S., Altanlar N. (2002). Synthesis of some new 2-substituted-phenyl-1*H*-benzimidazole-5-carbonitriles and their potent activity against *candida* species. Bioorg. Med. Chem..

[19] Penning T.D., Zhu G.D., Gandhi V.B., Gong J., Liu X., Shi Y., Klinghofer V., Johnson E.F., Donawho C.K., Frost D.J. (2009). Discovery of the Poly(ADP-ribose) Polymerase (PARP) Inhibitor 2-[(*R*)-2-methylpyrrolidin-2-yl]-1*H*-benzimidazole-4-carboxamide (ABT-888) for the Treatment of Cancer. J. Med. Chem..

[20] Abonia R., Cortes E., Insuasty B., Quiroga J., Nogueras M., Cobo J. (2011). Synthesis of novel 1,2,5-trisubstituted benzimidazoles as potential antitumor agents. Eur. J. Med. Chem..

[21] Demirayak S., Usama A., Mohsen A.C., Agri K. (2002). Synthesis and anticancer and anti-HIV testing of some pyrazino[1,2-a]benzimidazole derivatives. Eur. J. Med. Chem..

[22] Kamal A., Praveen Kumar P., Sreekanth K., Seshadri B.N., Ramulu P. (2008). Synthesis of new benzimidazole linked pyrrolo[2,1-c][1,4]benzodiazepine conjugates with efficient DNA- binding affinity and potent cytotoxicity. Bioorg. Med. Chem. Lett..

[23] Arumugam N., Almansour A.I., Suresh Kumar R., Perumal S., Ghabbour H.G., Fun H.-K. (2013). A 1,3-dipolar cycloaddition-annulation protocol for the expedient regio-, stereo- and product-selective construction of novel hybrid heterocycles comprising seven rings and seven contiguous stereocentres. Tetrahedron Lett..

[24] Almansour A.I., Arumugam N., Suresh Kumar R., Menéndez J.C., Ghabbour H.A., Fun H.-K., Ranjith Kumar R. (2015). Straightforward synthesis of pyrrolo[3,4-*b*]quinolines through intramolecular Povarov reactions. Tetrahedron Lett..

[25] Arumugam N., Raghunathan R., Almansour A.I., Karama U. (2012). An efficient synthesis of highly functionalized novel chromeno[4,3-*b*]pyrroles and indolizino[6,7-*b*]indoles as potent antimicrobial and antioxidant agents. Bioorg. Med. Chem. Lett..

[26] Arumugam N., Almansour A.I., Suresh Kumar R., Menéndez J.C., Sultan M.A., Karama U., Ghabbour H.A., Fun H.-K. (2015). An Expedient Regio- and Diastereoselective Synthesis of Hybrid Frameworks with Embedded Spiro[9,10]dihydroanthracene [9,3′]-pyrrolidine and Spiro[oxindole-3,2′-pyrrolidine] Motifs via an Ionic Liquid-Mediated Multicomponent Reaction. Molecules.

[27] Arumugam N., Abdul Rahim A.S., Hamid S.A., Osman H. (2012). Straightforward Synthesis of Novel 1-(2′-α-*O*-D-Glucopyranosyl ethyl) 2-Arylbenzimidazoles. Molecules.

[28] Abdul Rahim A.S., Salhimi S.M., Arumugam N., Pin L.C., Yee N.S., Muttiah N.N., Keat W.B., Hamid S.A., Osman H., Mat I.B. (2013). Microwave-assisted synthesis of *sec/tert*-butyl 2-arylbenzimidazoles and their unexpected antiproliferative activity towards ER negative breast cancer cells. J. Enzym. Inhib. Med. Chem..

[29] Meng L., Fettinger J.C., Kurth M.J. (2007). Intramolecular Cycloaddition of Azomethine Ylides in the Preparation of Pyrrolidino[2‘,3‘:3,4]pyrrolidino[1,2-*a*]benzimidazoles. Org. Lett..

[30] Butler P., Gallagher J.F., Manning A.R. (1998). Synthesis and characterisation of alkynylacetal and alkynylaldehyde derivatives of Ni(II) and Fe(II); the X-ray crystal structure of Ni(h5-C_5_H_5_)(PPh_3_)C-CCH(OEt)_2_. Inorg. Chem. Commun..

[B31-molecules-21-00724] 31.CCDC 1062849 (**9c**) and 974580 (**9d**) contains the supplementary crystallographic data for this paper. These data can be obtained free of charge via http://www.ccdc.cam.ac.uk/conts/retrieving.html (or from the CCDC, 12 Union Road, Cambridge CB2 1EZ, UK; Fax: +44 1223 336033; E-mail: deposit@ccdc.cam.ac.uk).

[32] Govindaraj J., Raja R., Suresh M., Raghunathan R., SubbiahPandi A. (2014). Crystal structures of methyl 3-phenyl-4,5-dihydro-1*H*,3*H*-benzo[4,5]imidazo[2,1-*c*][1,4]oxazepine-4-carboxylate and methyl 1-methyl-3-phenyl-4,5-dihydro-1*H*,3*H*-benzo[4,5]imidazo[2,1-*c*][1,4]oxazepine-4-carboxylate. Acta Crystallogr. Sect. E Struct. Rep. Online.

[B33-molecules-21-00724] 33.CCDC 1402796 contains the supplementary crystallographic data for this paper. These data can be obtained free of charge via http://www.ccdc.cam.ac.uk/conts/retrieving.html (or from the CCDC, 12 Union Road, Cambridge CB2 1EZ, UK; Fax: +44 1223 336033; E-mail: deposit@ccdc.cam.ac.uk).

[34] Murray J.S., Sen K. (1996). Molecular Electrostatic Potentials, Concepts and Applications.

[35] Scrocco E., Tomasi J. (1978). Electronic Molecular Structure, Reactivity and Intermolecular Forces: An Euristic Interpretation by Means of Electrostatic Molecular Potentials. Adv. Quantum Chem..

[36] Fukui K., Yonezawa T., Shingu H.J. (1952). A molecular-orbital theory of reactivity in aromatic hydrocarbons. J. Chem. Phys..

[37] Padmaja L., Ravikumar C., Sajan D., Joe I.H., Jayakumar V.S., Pettit G.R., Neilsen F.O. (2009). Density functional study on the structural conformations and intramolecular charge transfer from the vibrational spectra of the anticancer drug combretastatin-A2. J. Raman Spectrosc..

[38] Ravikumar C., Joe I.H., Jayakumar V.S. (2008). Charge transfer interactions and nonlinear optical properties of push-pull chromophore benzaldehyde phenylhydrazone: A vibrational approach. Chem. Phys. Lett..

[39] Geskin V.M., Lambert C., Bredas J.-L. (2003). Origin of High Second- and Third-Order Nonlinear Optical Response in Ammonio/Borato Diphenylpolyene Zwitterions: The Remarkable Role of Polarized Aromatic Groups. J. Am. Chem. Soc..

[40] Pu L.S. (1991). Observing High Second Harmonic Generation and Control of Molecular Alignment in One Dimension Cyclobutenediones as a Promising New Acceptor for Nonlinear Optical Materials. Materials for Nonlinear Optics.

[41] Frisch M.J., Trucks G.W., Schlegel H.B., Scuseria G.E., Robb M.A., Cheeseman J.R., Montgomery J.A., Vreven T., Kudin K.N., Burant J.C. (2004). Gaussian—03, Revision C.01.

[42] Dennington R., Keith T., Millam J. (2007). GaussView, Version 4.1.

