# Supplementary material for: Synthesis, Spectroscopic, X-ray Diffraction and DFT Studies of Novel Benzimidazole Fused-1,4-Oxazepines
Source: Molecules. 2016 Jun 3;21(6):724. doi: 10.3390/molecules21060724 (PMC6272878; doi:10.3390/molecules21060724)
Supplement: Supplementary file 1 [file molecules-21-00724-s001.pdf]

Supplementary Materials: Synthesis, Spectroscopic, X-ray Diffraction and DFT Studies of Novel Benzimidazole-Fused 1,4-oxazepines

Abdulrahman I. Almansour, Natarajan Arumugam, Raju Suresh Kumar, Saied M. Soliman, Mohammad Altaf and Hazem A. Ghabbour

Supporting Information

S. No.	Figure	Page
1	^{1}H-NMR spectrum of **5**	**S5**
2	^{13}C-NMR spectrum of **5**	**S6**
3	^{1}H-NMR spectrum of **6**	**S7**
4	^{13}C-NMR spectrum of **6**	**S8**
5	^{1}H-NMR spectrum of **9e**	**S9**
6	^{13}C-NMR spectrum of **9e**	**S10**
7	^{1}H-NMR spectrum of **10e**	**S11**
8	^{13}C-NMR spectrum of **10e**	**S12**

Table 1. X-ray crystallography experimental details.

	**9c**	**9d**	**10b**
**Crystal Data**			
Chemical formula	$C_{19}H_{20}ClN_3O$	$C_{19}H_{20}ClN_3O$	$C_{19}H_{20}BrN_3O$
M_r	341.83	341.83	386.29
Crystal system, space group	Orthorhombic, *Pca*2$_1$	Triclinic, *P-1*	Triclinic, *P*-1
Temperature (K)	100	293	100
a, *b*, *c* (Å)	18.3707 (5), 6.3836 (2), 15.0003 (4)	9.2348 (6), 9.2539 (6), 11.0572 (8)	9.7315 (4), 13.5364 (5), 13.8981 (6)
α, β, γ (°)	90.00	88.458 (4), 74.006 (4), 75.498 (3)	71.842 (1), 83.852 (2), 79.480 (2)
V (Å^3)	1759.10 (9)	878.51 (10)	1707.91 (12)
Z	4	2	4
Radiation type	Mo *K*α	Mo Kα	Mo *K*α
μ (mm^{-1})	0.23	0.23	2.42
Crystal size (mm)	0.37 × 0.23 × 0.20	0.30 × 0.25 × 0.20	0.59 × 0.34 × 0.29
**Data Collection**			
Diffractometer	Bruker *APEX*-II D8 venture diffractometer	Bruker Kappa *APEX*-II diffractometer	Bruker *APEX*-II D8 Venture diffractometer
Absorption correction	Multi-scan *SADABS* V2014/3 (Bruker AXS Inc., Karlsruhe, Germany)	Multi-scan (*SADABS*; Sheldrick, 1996)	Multi-scan *SADABS* Bruker 2014
T_{min}, T_{max}	0.92, 0.95	0.935, 0.956	0.328, 0.544
No. of measured, independent and observed [**I > 2σ(I)**] reflections	44,092, 7028, 6737	12,932, 3593, 2899	42,167, 9941, 6814
R_{int}	0.026	0.023	0.077
$R[F^2 > 2\sigma(F^2)]$, $wR(F^2)$, S	0.025, 0.071, 1.04	0.040, 0.108, 1.06	0.046, 0.096, 1.00
No. of reflections	7028	3593	9941
No. of parameters	223	220	464
No. of restraints	1	0	0
H-atom treatment	H atoms treated by a mixture of independent and constrained refinement	H atoms treated by a mixture of independent and constrained refinement	H atoms treated by a mixture of independent and constrained refinement
$\Delta\rho_{max}$, $\Delta\rho_{min}$ (e Å^{-3})	0.28, −0.21	0.28, −0.35	0.69, −0.89
CCDC number	1,062,849	974,580	1,402,796

Table 2. The experimental and calculated geometric parameters of the studied compound using DFT B3LYP/6–31 G(d,p) method.

Parameter	Calc.	Exp	Parameter	Calc.	Exp	Parameter	Calc.	Exp
	**9c**			**9d**			**10b**	
R(1–7)	1.757	1.739	R(1–2)	1.400	1.387	R(1–6)	1.919	1.909
R(2–36)	1.442	1.433	R(1–10)	1.419	1.397	R(2–16)	1.443	1.435
R(4–16)	1.401	1.412	R(1–39)	1.380	1.374	R(2–35)	1.445	1.462
R(4–17)	1.285	1.278	R(2–4)	1.390	1.369	R(3–15)	1.378	1.380
R(5–19)	1.328	1.332	R(4–6)	1.414	1.400	R(3–16)	1.419	1.422
R(5–20)	1.373	1.385	R(6–8)	1.387	1.363	R(4–18)	1.311	1.315
R(6–19)	1.386	1.368	R(8–10)	1.405	1.399	R(4–19)	1.386	1.388
R(6–29)	1.38	1.382	R(10–40)	1.373	1.379	R(5–18)	1.377	1.359
R(6–30)	1.465	1.467	R(11–12)	1.451	1.453	R(5–28)	1.390	1.390
R(7–8)	1.392	1.388	R(11–39)	1.386	1.367	R(5–29)	1.458	1.469
R(7–16)	1.409	1.401	R(11–40)	1.328	1.324	R(6–7)	1.389	1.378
R(8–10)	1.395	1.390	R(12–41)	1.286	1.257	R(6–15)	1.412	1.396
R(10–12)	1.395	1.389	R(14–15)	1.405	1.382	R(7–9)	1.395	1.390
R(12–14)	1.392	1.392	R(14–22)	1.406	1.388	R(9–11)	1.394	1.381
R(14–16)	1.408	1.399	R(14–41)	1.406	1.414	R(11–13)	1.392	1.382
R(17–19)	1.451	1.460	R(15–17)	1.391	1.375	R(13–15)	1.409	1.402
R(20–21)	1.406	1.402	R(17–19)	1.396	1.369	R(16–18)	1.513	1.509
R(20–29)	1.419	1.408	R(19–20)	1.394	1.371	R(19–20)	1.400	1.401
R(21–23)	1.387	1.384	R(19–42)	1.758	1.737	R(19–28)	1.415	1.406
R(23–25)	1.415	1.411	R(20–22)	1.393	1.384	R(20–22)	1.390	1.379
R(25–27)	1.389	1.385	R(24–27)	1.536	1.515	R(22–24)	1.409	1.402
R(27–29)	1.401	1.397	R(24–39)	1.464	1.463	R(24–26)	1.392	1.381
R(30–33)	1.536	1.522	R(27–30)	1.539	1.522	R(26–28)	1.397	1.390
R(33–36)	1.539	1.535	R(30–31)	1.536	1.515	R(29–32)	1.533	1.526
R(36–37)	1.536	1.525	R(30–35)	1.535	1.515	R(32–35)	1.549	1.528
R(36–41)	1.535	1.528	R(30–43)	1.441	1.420	R(35–36)	1.534	1.525
						R(35–40)	1.540	1.530
RMSD (R^2)	0.009 (0.997)			0.018 (0.994)			0.010 (0.997)	
A(1-7-8)	118.6	118.2	A(2-1-10)	122.5	122.3	A(1-6-7)	118.4	118.5
A(1-7-16)	119.9	120.1	A(2-1-39)	131.9	131.6	A(1-6-15)	119.4	118.8
A(2-36-33)	104.7	105.4	A(1-2-4)	116.5	116.6	A(16-2-35)	121.0	118.5
A(2-36-37)	109.8	110.2	A(10-1-39)	105.7	106.1	A(2-16-3)	109.4	109.6
A(2-36-41)	109.4	110.0	A(1-10-8)	119.9	120.1	A(2-16-17)	108.6	110.2
A(16-4-17)	119.2	118.4	A(1-10-40)	110.1	109.6	A(2-16-18)	111.0	110.2
A(4-16-7)	120.0	119.1	A(1-39-11)	105.9	106.4	A(2-35-32)	112.1	111.9
A(4-16-14)	122.5	122.9	A(1-39-24)	124.4	123.9	A(2-35-36)	102.8	102.8
A(4-17-19)	124.5	122.4	A(2-4-6)	121.9	121.8	A(2-35-40)	112.2	112.0
A(19-5-20)	105.1	104.8	A(4-6-8)	121.4	121.8	A(15-3-16)	125.5	126.0
A(5-19-6)	113.2	113.2	A(6-8-10)	117.9	117.5	A(15-3-44)	118.8	118.2
A(5-19-17)	119.9	120.2	A(8-10-40)	130.0	130.3	A(3-15-6)	120.8	120.6
A(5-20-21)	130.0	130.2	A(10-40-11)	105.1	105.2	A(3-15-13)	122.6	122.6
A(5-20-29)	110.1	109.7	A(12-11-39)	126.8	125.9	A(16-3-44)	115.3	115.6
A(19-6-29)	105.9	106.3	A(12-11-40)	120.0	121.3	A(3-16-17)	110.7	110.2
A(19-6-30)	129.8	129.6	A(11-12-41)	124.4	123.7	A(3-16-18)	107.4	106.3
A(6-19-17)	126.9	126.4	A(39-11-40)	113.1	112.7	A(18-4-19)	105.1	104.1
A(29-6-30)	124.4	124.1	A(11-39-24)	129.7	129.6	A(4-18-5)	113.7	114.4
A(6-29-20)	105.7	106.0	A(12-41-14)	119.6	120.9	A(4-18-16)	124.5	124.4
A(6-29-27)	131.8	131.4	A(15-14-22)	118.7	118.2	A(4-19-20)	129.9	130.3
A(6-30-33)	112.2	111.0	A(15-14-41)	117.6	116.5	A(4-19-28)	110.0	110.4
A(8-7-16)	121.5	121.7	A(14-15-17)	121.0	121.6	A(18-5-28)	106.2	106.3
A(7-8-10)	119.9	119.4	A(22-14-41)	123.7	125.2	A(18-5-29)	126.4	126.1
A(7-16-14)	117.4	117.9	A(14-22-20)	120.7	120.5	A(5-18-16)	121.8	121.2
A(8-10-12)	119.8	120.0	A(15-17-19)	119.2	119.3	A(28-5-29)	127.3	127.5
A(10-12-14)	120.1	120.3	A(17-19-42)	119.5	118.9	A(5-28-19)	105.0	104.9
A(12-14-16)	121.3	120.7	A(20-19-42)	119.5	120.4	A(5-28-26)	132.8	132.2
A(21-20-29)	119.9	120.0	A(19-20-22)	119.4	119.8	A(5-29-30)	109.6	109.2
A(20-21-23)	117.9	117.7	A(27-24-39)	112.1	110.4	A(5-29-31)	106.9	109.3
A(20-29-27)	122.5	122.6	A(24-27-30)	113.6	114.0	A(5-29-32)	112.6	111.8
A(21-23-25)	121.4	121.6	A(27-30-31)	112.4	111.7	A(7-6-15)	122.2	122.7
A(23-25-27)	121.9	121.6	A(27-30-35)	109.9	109.6	A(6-7-9)	120.1	119.4

A(25-27-29)	116.5	116.6	A(27-30-43)	104.6	105.7	A(6-15-13)	116.5	116.8
A(30-33-36)	113.5	113.5	A(31-30-35)	110.6	110.1	A(7-9-11)	118.9	119.0
A(33-36-37)	112.4	112.0	A(31-30-43)	109.8	109.6	A(9-11-13)	120.9	121.3
A(33-36-41)	109.8	108.5	A(35-30-43)	109.4	110.0	A(11-13-15)	121.4	120.7
A(37-36-41)	110.6	110.6	A(2-1-10)	122.5	122.3	A(20-19-28)	120.1	119.3
						A(19-20-22)	118.0	118.1
						A(19-28-26)	122.2	123.0
						A(20-22-24)	121.3	121.5
						A(22-24-26)	121.6	121.7
						A(24-26-28)	116.8	116.5
						A(29-32-35)	117.1	117.4
						A(32-35-36)	108.6	108.9
						A(32-35-40)	111.5	111.6
						A(36-35-40)	109.2	109.2
RMSD (R^2)	0.4 (0.997)			0.7 (0.995)			0.7 (0.995)	

Table 3. The natural atomic charges calculated at the B3LYP/6-31G(d,p)

Atom	NAC	Atom	NAC	Atom	NAC
**9c**		**9d**		**10b**	
Cl1	−0.0025	C1	0.1469	Br1	0.0453
O2	−0.7778	C2	−0.2757	O2	−0.6020
H3	0.4779	H3	0.2537	N3	−0.6350
N4	−0.4553	C4	−0.2291	N4	−0.5041
N5	−0.4584	H5	0.2415	N5	−0.3987
N6	−0.3704	C6	−0.2570	C6	−0.1397
C7	−0.0414	H7	0.2415	C7	−0.2372
C8	−0.2454	C8	−0.2231	H8	0.2546
H9	0.2575	H9	0.2495	C9	−0.2694
C10	−0.2346	C10	0.1127	H10	0.2420
H11	0.2466	C11	0.3412	C11	−0.2220
C12	−0.2326	C12	0.0715	H12	0.2391
H13	0.2462	H13	0.2161	C13	−0.2807
C14	−0.2482	C14	0.1292	H14	0.2352
H15	0.2485	C15	−0.2192	C15	0.1701
C16	0.1192	H16	0.2500	C16	0.2292
C17	0.0782	C17	−0.2478	H17	0.2177
H18	0.2184	H18	0.2578	C18	0.4050
C19	0.3396	C19	−0.0478	C19	0.1193
C20	0.1125	C20	−0.2438	C20	−0.2253
C21	−0.2231	H21	0.2587	H21	0.2508
H22	0.2492	C22	−0.2461	C22	−0.2561
C23	−0.2570	H23	0.2487	H23	0.2417
H24	0.2413	C24	−0.2655	C24	−0.2374
C25	−0.2289	H25	0.2545	H25	0.2409
H26	0.2415	H26	0.2682	C26	−0.2736
C27	−0.2748	C27	−0.4902	H27	0.2374
H28	0.2551	H28	0.2497	C28	0.1356
C29	0.1478	H29	0.2507	C29	−0.2597
C30	−0.2653	C30	0.2898	H30	0.2402
H31	0.2534	C31	−0.7137	H31	0.2567
H32	0.2683	H32	0.2430	C32	−0.5039
C33	−0.4922	H33	0.2411	H33	0.2508
H34	0.2576	H34	0.2310	H34	0.2565
H35	0.2488	C35	−0.7068	C35	0.2818
C36	0.2903	H36	0.2301	C36	−0.6917
C37	−0.7162	H37	0.2392	H37	0.2482
H38	0.2407	H38	0.2453	H38	0.2497
H39	0.2494	N39	−0.3728	H39	0.2336
H40	0.2287	N40	−0.4592	C40	−0.7191
C41	−0.7061	N41	−0.4620	H41	0.2305
H42	0.2290	Cl42	−0.0046	H42	0.2455
H43	0.2409	O43	−0.7761	H43	0.2465

Table 4. The hyperpolarizability β_0 (a.u.) of the studied compounds.

Parameter	Urea	9c	9d	10b
β_{xyy}	0.014	−2317.995	952.389	−70.608
β_{yyy}	36.648	−105.860	49.949	187.152
β_{xxz}	−0.004	15.573	−15.171	119.083
β_{xyz}	−90.412	−59.186	54.322	173.580
β_{yyz}	0.017	−73.529	146.965	−88.587
β_{xzz}	13.171	70.365	65.717	23.381
β_{yzz}	0.017	81.306	−7.738	−51.112
β_{zzz}	−0.004	1.426	3.595	−0.862
β_{xyy}	−16.150	−10.243	13.443	12.168
β_{yyy}	0.014	19.402	−0.471	−18.552
β_x	0.006	−2300.996	940.813	47.613
β_y	−69.914	−175.290	117.714	372.900
β_z	0.047	27.179	138.756	−158.251
β_0	69.914	2307.823	958.248	407.879

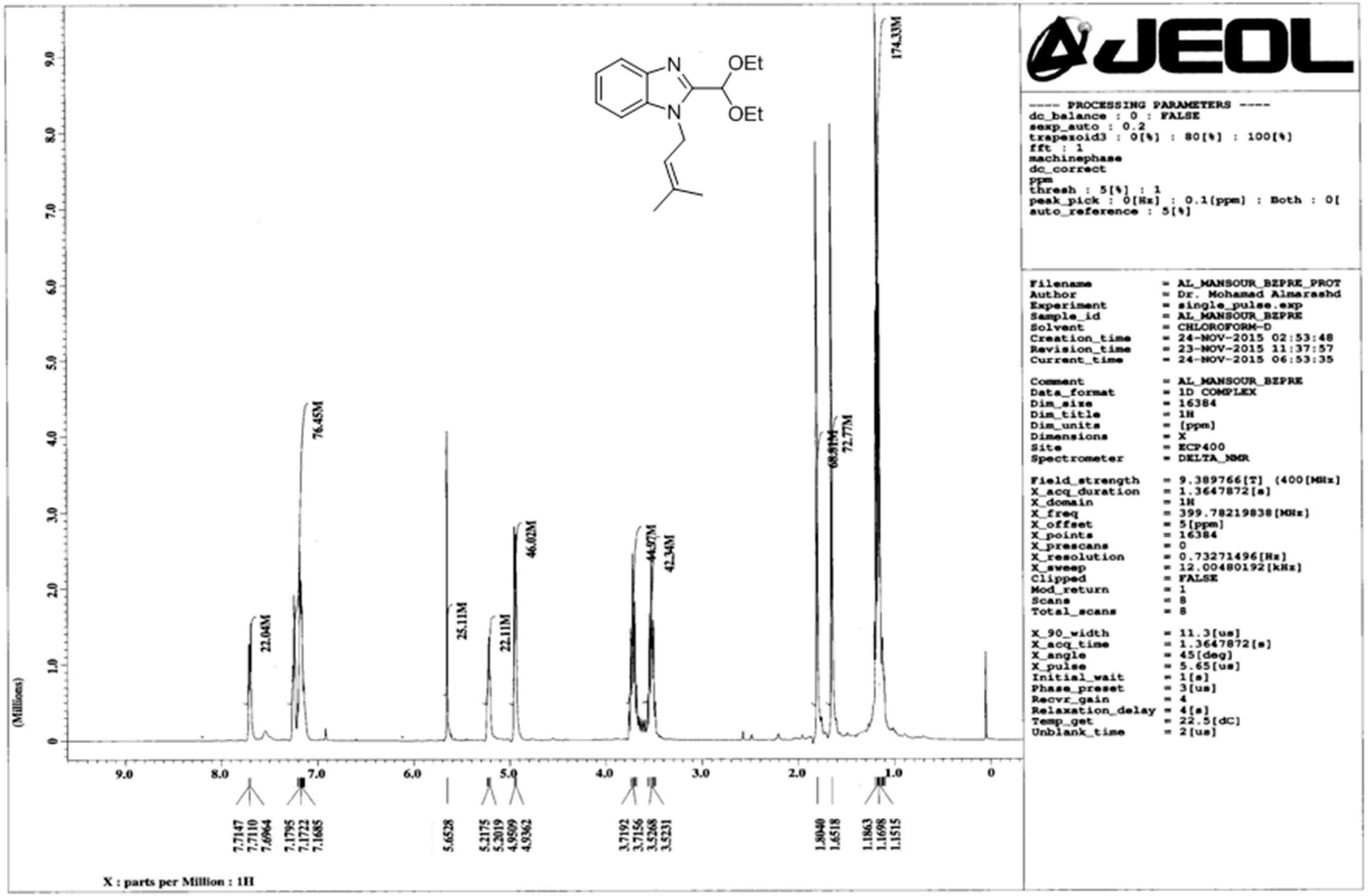

Figure 1. ^{1}H-NMR spectrum of **5**.

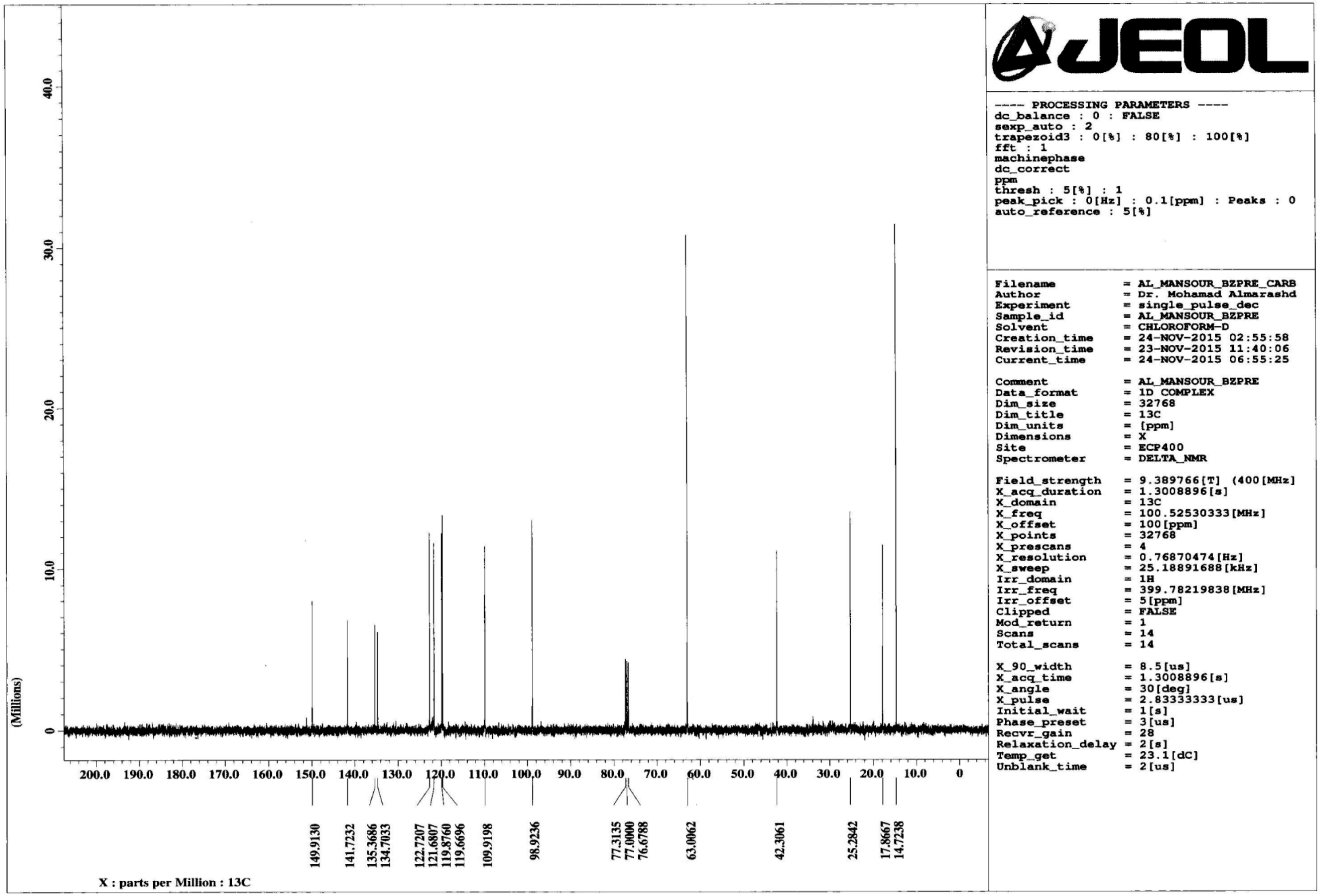

Figure 2. ^{13}C-NMR spectrum of **5**.

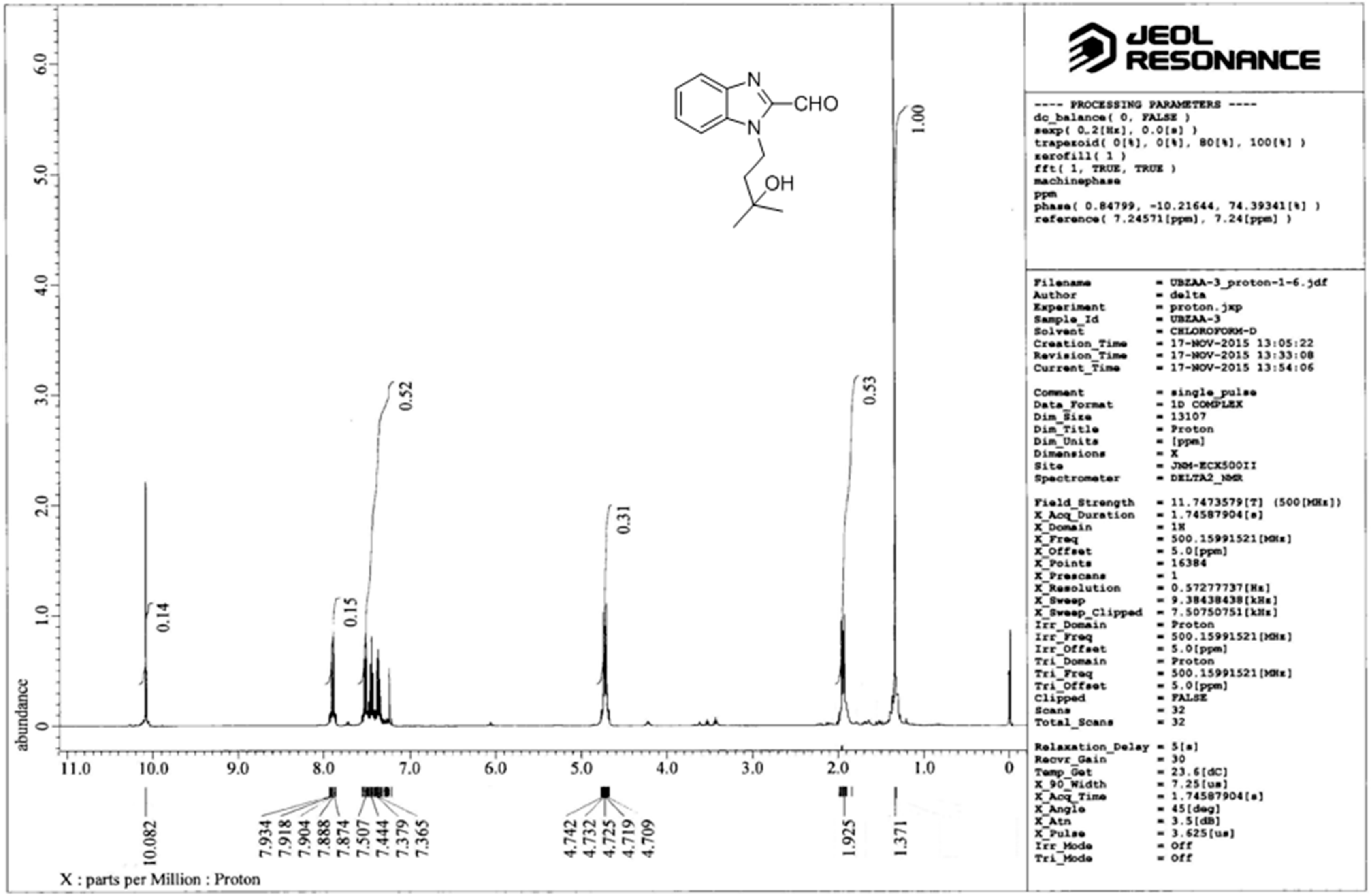

Figure 3. ^{1}H-NMR spectrum of **6**.

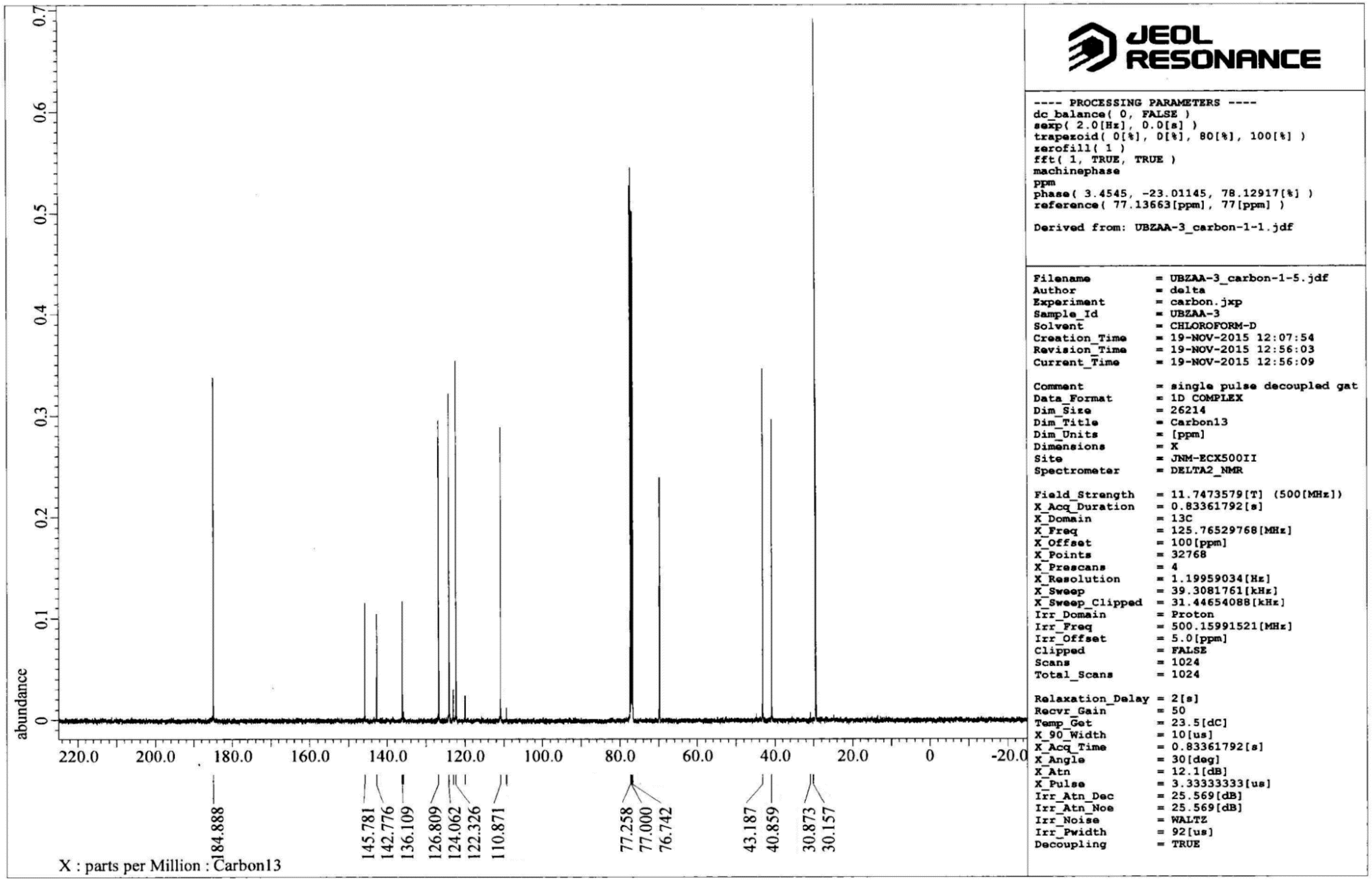

Figure 4. ^{13}C-NMR spectrum of **6**.

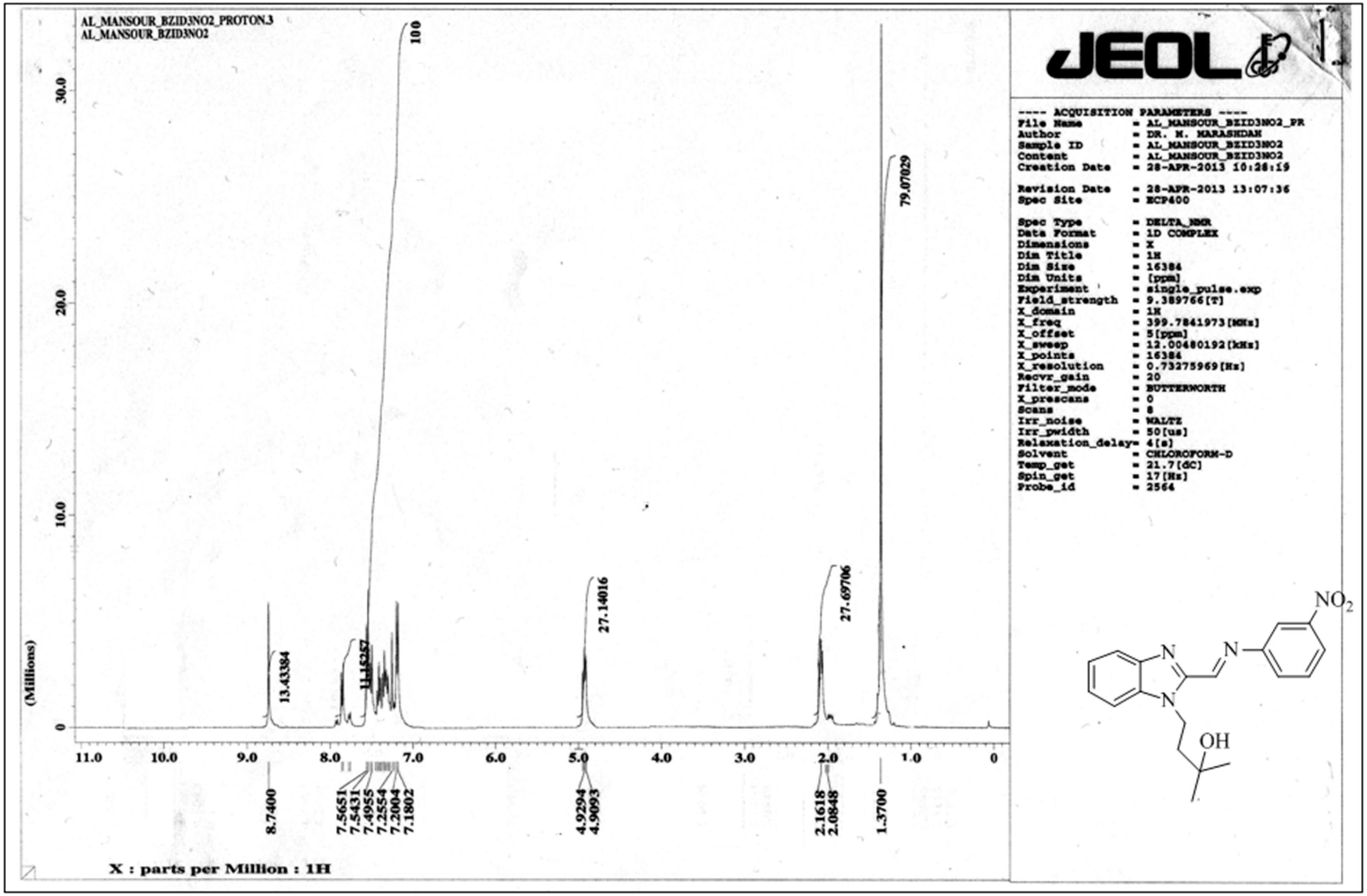

Figure 5. ^{1}H-NMR spectrum of **9e**.

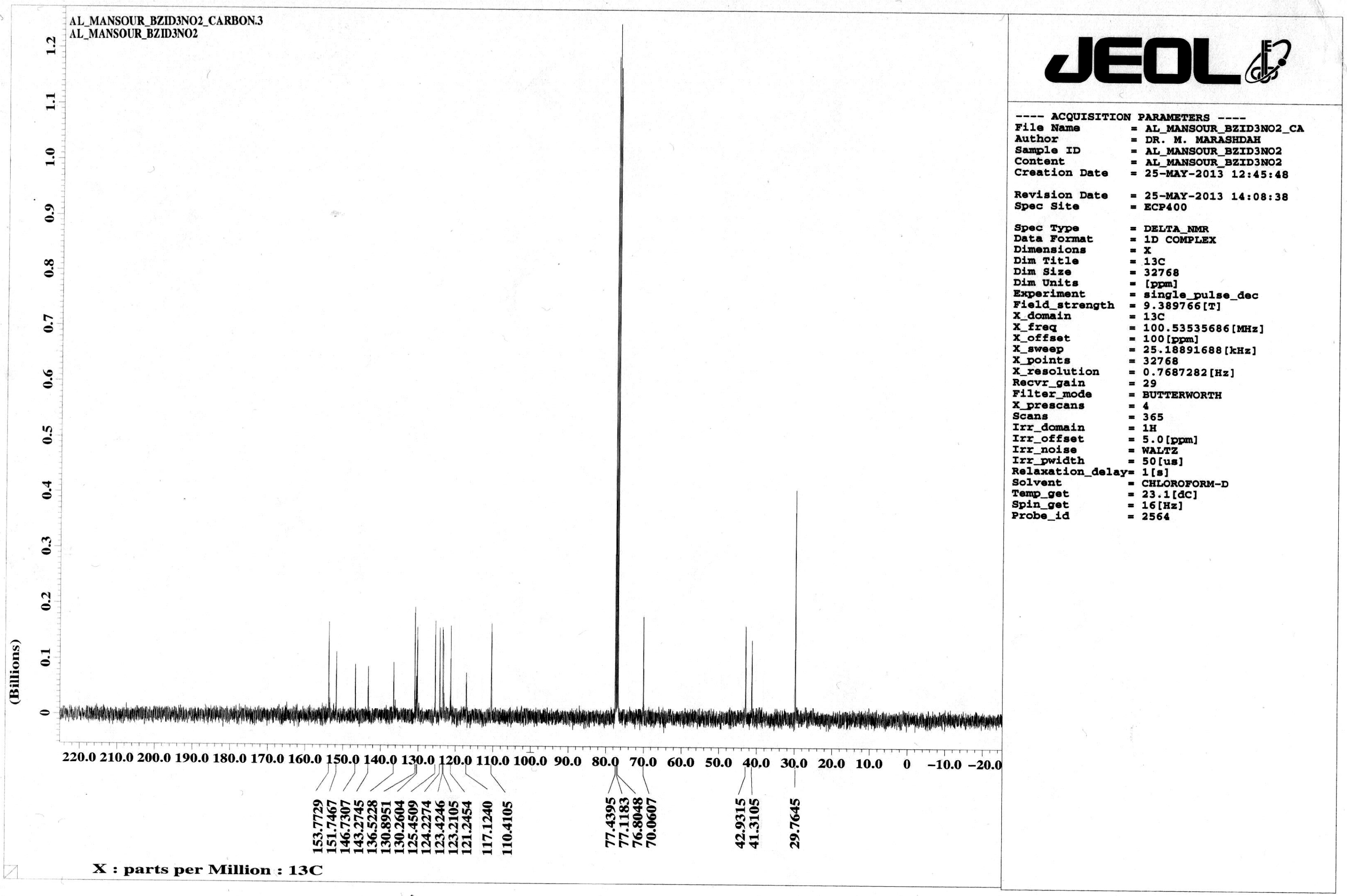

Figure 6. ^{13}C-NMR spectrum of **9e**.

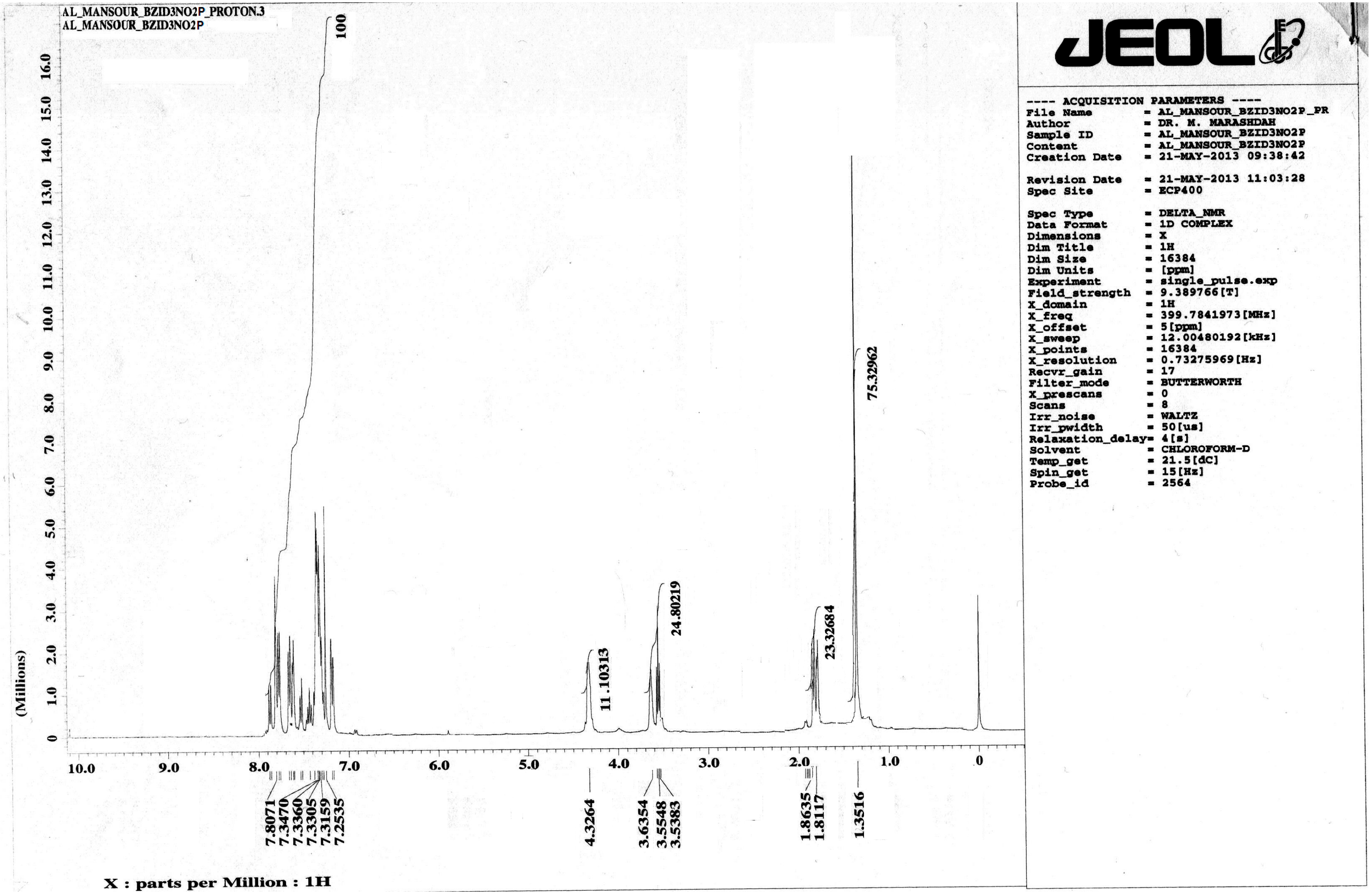

Figure 7. ^{1}H-NMR spectrum of **10e**.

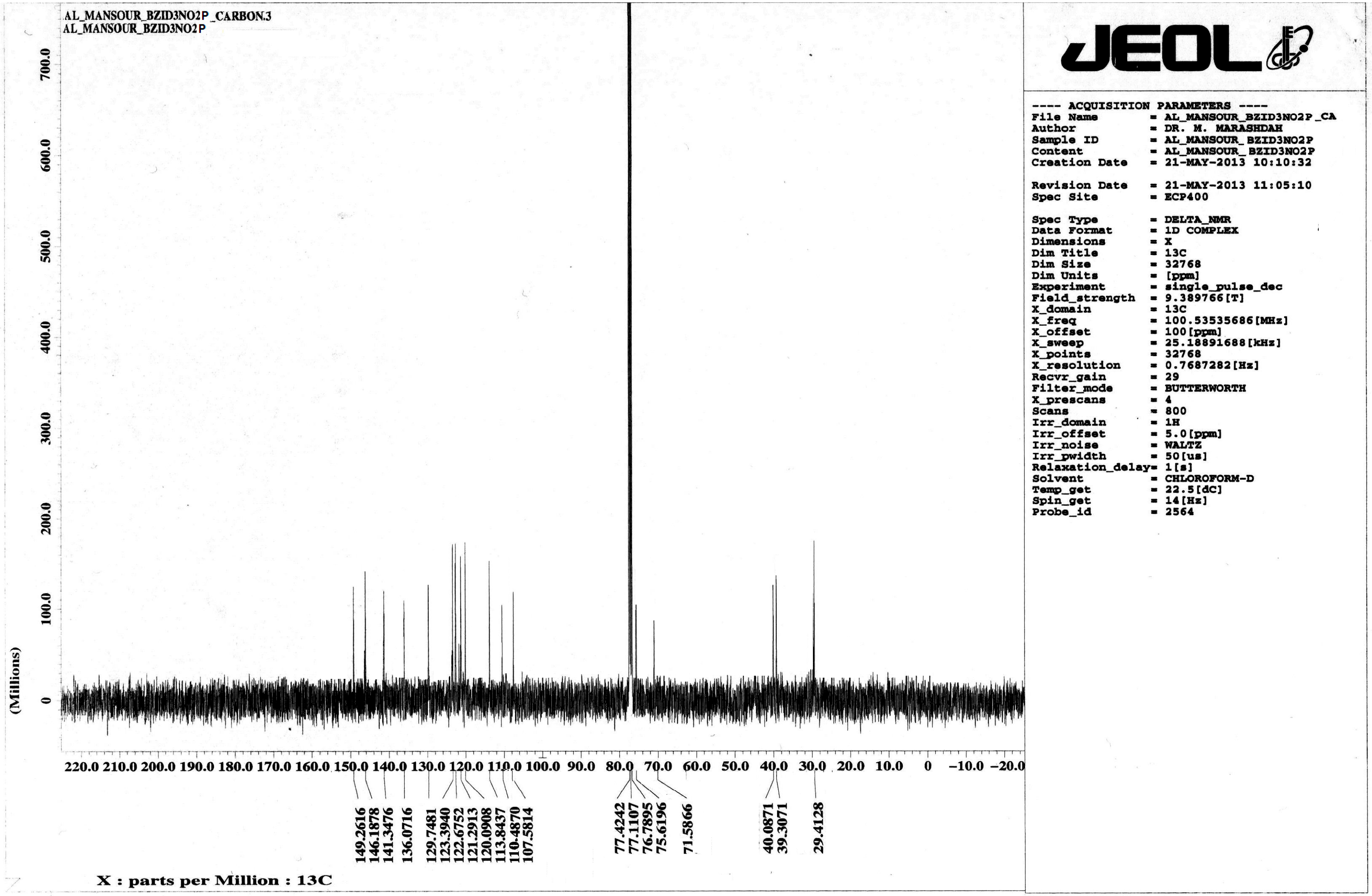

Figure 8. ^{13}C-NMR spectrum of **10e**.
